# HMGB1 Attenuates Cardiac Remodelling in the Failing Heart via Enhanced Cardiac Regeneration and miR-206-Mediated Inhibition of TIMP-3

**DOI:** 10.1371/journal.pone.0019845

**Published:** 2011-06-22

**Authors:** Federica Limana, Grazia Esposito, Daniela D'Arcangelo, Anna Di Carlo, Sveva Romani, Guido Melillo, Antonella Mangoni, Chiara Bertolami, Giulio Pompilio, Antonia Germani, Maurizio C. Capogrossi

**Affiliations:** 1 Laboratorio di Biologia Vascolare e Medicina Rigenerativa, Centro Cardiologico Monzino-Istituto di Ricovero e Cura a Carattere Scientifico (IRCCS), Milan, Italy; 2 Laboratorio di Patologia Vascolare, Istituto Dermopatico dell'Immacolata-IRCCS, Rome, Italy; 3 Mendel Laboratory, Casa Sollievo della Sofferenza-IRCCS, San Giovanni Rotondo, Italy; 4 Fondazione Livio Patrizi, Rome, Italy; Brigham and Women's Hospital, United States of America

## Abstract

**Aims:**

HMGB1 injection into the mouse heart, acutely after myocardial infarction (MI), improves left ventricular (LV) function and prevents remodeling. Here, we examined the effect of HMGB1 in chronically failing hearts.

**Methods and Results:**

Adult C57 BL16 female mice underwent coronary artery ligation; three weeks later 200 ng HMGB1 or denatured HMGB1 (control) were injected in the peri-infarcted region of mouse failing hearts. Four weeks after treatment, both echocardiography and hemodynamics demonstrated a significant improvement in LV function in HMGB1-treated mice. Further, HMGB1-treated mice exhibited a ∼23% reduction in LV volume, a ∼48% increase in infarcted wall thickness and a ∼14% reduction in collagen deposition. HMGB1 induced cardiac regeneration and, within the infarcted region, it was found a ∼2-fold increase in c-kit^+^ cell number, a ∼13-fold increase in newly formed myocytes and a ∼2-fold increase in arteriole length density. HMGB1 also enhanced MMP2 and MMP9 activity and decreased TIMP-3 levels. Importantly, miR-206 expression 3 days after HMGB1 treatment was 4-5-fold higher than in control hearts and 20–25 fold higher that in sham operated hearts. HMGB1 ability to increase miR-206 was confirmed *in vitro*, in cardiac fibroblasts. TIMP3 was identified as a potential miR-206 target by TargetScan prediction analysis; further, in cultured cardiac fibroblasts, miR-206 gain- and loss-of-function studies and luciferase reporter assays showed that TIMP3 is a direct target of miR-206.

**Conclusions:**

HMGB1 injected into chronically failing hearts enhanced LV function and attenuated LV remodelling; these effects were associated with cardiac regeneration, increased collagenolytic activity, miR-206 overexpression and miR-206 -mediated inhibition of TIMP-3.

## Introduction

Although in recent years post-infarction survival rates have improved, left ventricular failure due to loss of cardiomyocytes and remodeling remains a major health problem; it has a poor prognosis and represents the most frequent cause of hospitalization among elderly adult patients. Current treatments that result in definite clinical benefits in humans are represented by prompt reperfusion of the ischemic myocardium and pharmacologic therapy mostly based on the inhibition of the renin-angiotensin-aldosterone axis and the sympathetic nervous system. It is noteworthy that these treatments have no effect on contractile mass loss [1].

In the last years efforts have been performed to promote cardiac tissue regeneration with bone marrow [2] and, recently, with cardiac stem cell (CSC) transplantation [3]. Further, in animal models, it has been shown that it is possible to promote resident CSC proliferation and differentiation *in vivo,* by injecting cytokines or growth factors directly into the heart either in the acute [4], [5] and chronic [6] phase following infarction.

High Mobility Group Box-1 protein (HMGB1) is a highly conserved nuclear protein that acts as a chromatin-binding factor capable of promoting access of transcriptional complexes to the DNA. In addition to its nuclear role, HMGB1 functions as an extracellular signalling molecule regulating both inflammation and regenerating processes [7]. In presence of tissue damage, both inflammatory and necrotic cells release HMGB1 and the extracellular protein stimulates monocytes/macrophages and neutrophils to secrete inflammatory cytokines amplifying the inflammatory response. Further, in different *in vivo* models of human diseases HMGB1 stimulates tissue repair [8]. Our laboratory has shown that HMGB1 administration, acutely after myocardial infarction, induces cardiac progenitor cell proliferation and differentiation, myocardial regeneration and an improvement in cardiac performance. This result is in agreement with other studies which have examined HMGB1 ability to activate vessel associated stem cells [9], endothelial progenitor cells (EPCs) [10] and myogenic cells [11]. Taken together these results raise the possibility that exogenous HMGB1 may be used to activate resident stem cells and may have a therapeutic action that would provide an alternative to cell transplantation. However, other mechanisms which may account for HMGB1 therapeutic potential in the context of ischemic cardiac damage are still poorly characterized. In the present work we examined the effect of exogenous HMGB1 in a murine model of heart failure and found that HMGB1 intramyocardial injection improved left ventricular (LV) function and remodelling; these effects were associated with cardiac regeneration, increased collagenolytic activity, miR-206 overexpression and miR-206 -mediated inhibition of tissue inhibitor of metalloproteinase 3 (TIMP-3).

## Results

### HMGB1 improves cardiac function, left ventricular remodelling and mouse survival

We first examined whether HMGB1 injected into the LV wall of failing hearts, three weeks after MI, had an effect on LV function and remodelling, and on animal survival (Figure S1).

**Figure 1:**
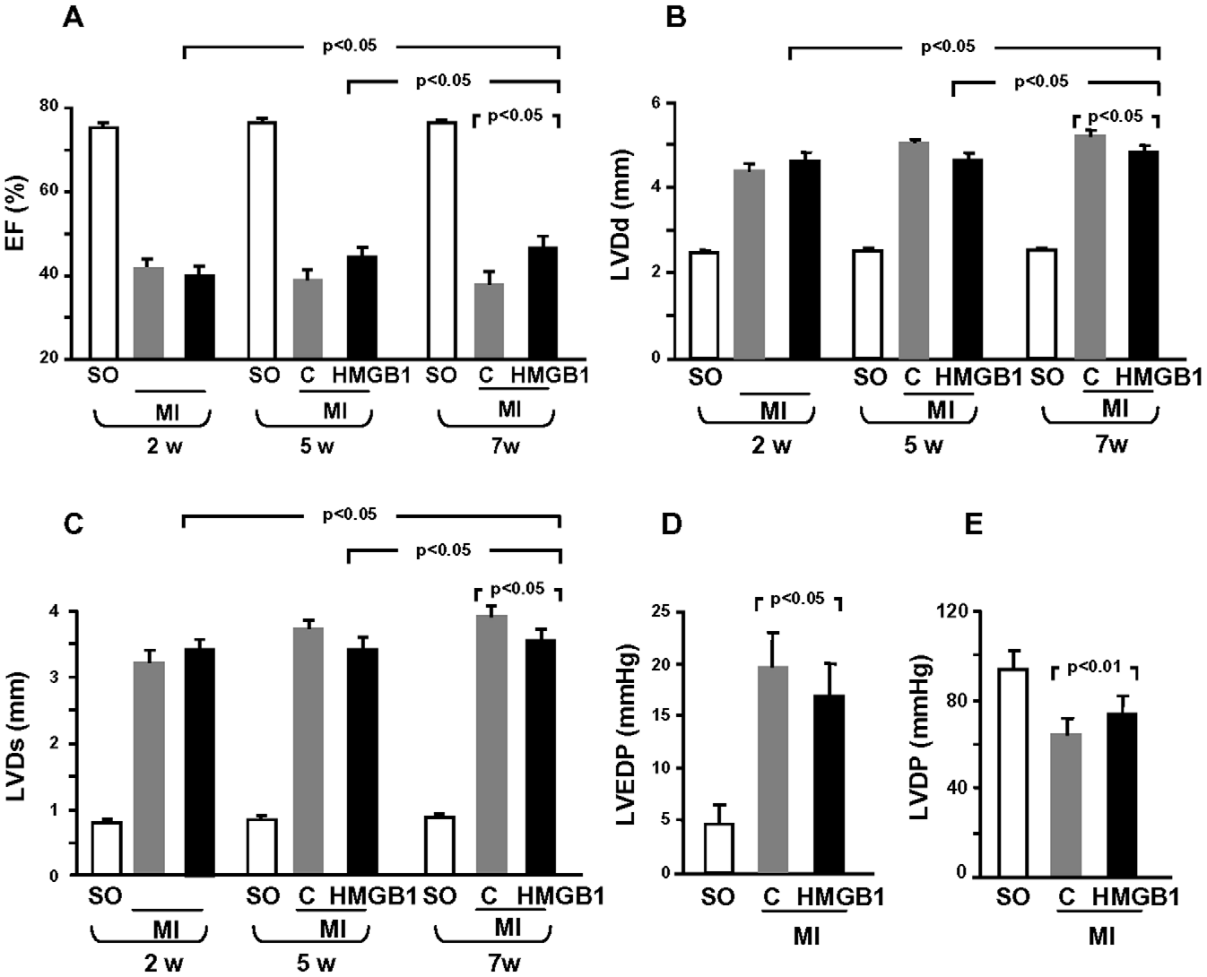
Echocardiography and hemodynamic studies after MI. Both echocardiographic and hemodynamic studies were performed to evaluate LV function and size in infarcted mice (MI) treated with HMGB1 (HMGB1) or with denatured HMGB1 (control; C), and in sham operated untreated mice (SO). (A–C) Echocardiography. LV ejection fraction (EF), LV diameter in diastole (LVDd) and LV diameter in systole (LVDs) were measured 2 weeks after MI, before treatment, and again 5 and 7 weeks following MI, i.e. 2 and 4 weeks after HMGB1 or denatured HMGB1 injection (SO, n = 10; control, n = 10; HMGB1, n = 15). HMGB1 treatment improved EF and inhibited the progressive increase in LV dilation. (D,E) Hemodynamic measurements were performed 7 weeks after MI, just before sacrifice. HMGB1 treatment ameliorated LV end-diastolic pressure (LVEDP) and LV developed pressure (LVDP) (SO, n = 10; control, n = 14; HMGB1, n = 19). Results are presented as mean±standard deviation.

Echocardiographic studies were performed two weeks after MI, i.e. one week prior to HMGB1 injection, and repeated 2 and 4 weeks following treatment. At the first time point, Ejection Fraction (EF) was markedly lower (Figure 1A) and LV diameter in diastole (LVDd) and in systole (LVDs) were higher (Figures 1B and 1C) in infarcted mice compared to sham operated animals; importantly, infarcted mice, which were subsequently randomized either to control or HMGB1 treatment, displayed similar echocardiographic parameters.

HMGB1-injected animals exhibited a progressive increase in EF whereas LV function of control mice progressively deteriorated and, 4 weeks after treatment, the two groups were significantly different (Figure 1A). Similarly, LVDd and LVDs progressively increased in control whereas LV dilation was prevented in HMGB1-treated animals (Figures 1B and C). It is noteworthy that all echocardiographic measurements demonstrated an improvement not only in HMGB1-treated mice compared to untreated mice but also in HMGB1-treated animals, at the different time points of the analysis (Figures 1A–C).

**Figure 2:**
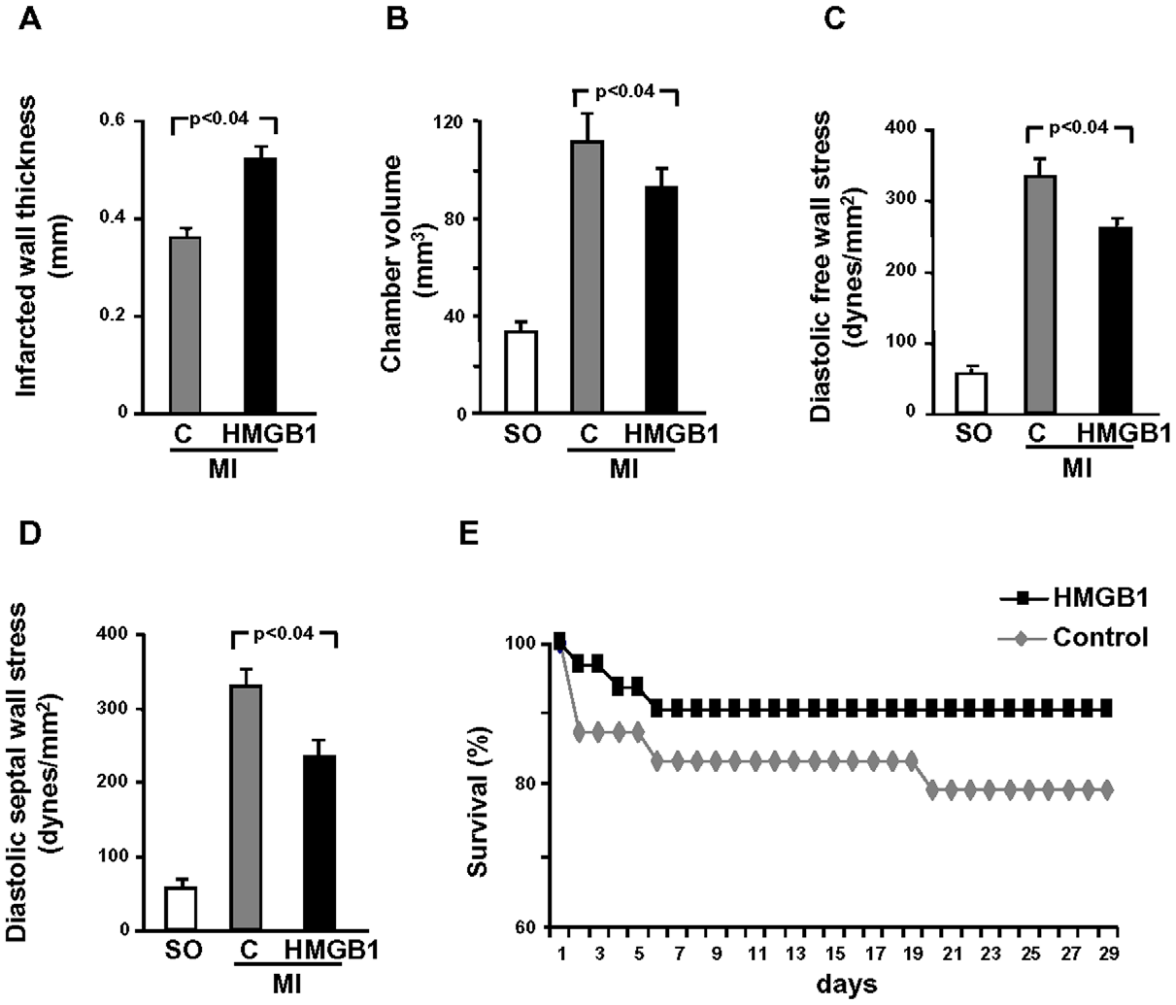
Cardiac anatomy, wall stress and animal survival after MI. Infarcted wall thickness was enhanced in HMGB1 vs control mice (A). Further, HMGB1 attenuated the increase in LV volume (B), diastolic free wall stress (C) and diastolic septal wall stress (D) following MI. All measurements were obtained 4 weeks after HMGB1 or control treatment (Results are presented as mean±standard deviation; SO, n = 10; control, n = 14; HMGB1, n = 18). (F) HMGB1 improved survival vs control during the 4 weeks following treatment (control, n = 24; HMGB1, n = 31; log-rank test, p<0.002).

Hemodynamic measurements were performed 4 weeks after treatment, prior to sacrifice, and, in agreement with the echocardiograpic studies, confirmed the beneficial effect of HMGB1 treatment. Specifically, HMGB1 attenuated the increase in LV end-diastolic pressure (LVEDP; Figure 1D) and the decrease in LV developed pressure (LVDP; Figure 1E); in contrast, the effect on ±dP/dt was marginal and did not achieve statistical significance (Figure S2). Further, the functional improvement in HMGB1-treated hearts was associated with a thicker infarcted wall and reduced LV chamber dilation (Figures 2A and 2B). The attenuation in ventricular dilation together with the hemodynamic parameters resulted in a reduction in diastolic free wall and septal wall stress (Figures 2C and 2D).

Finally, the above functional and morphometric changes in HMGB1-treated animals resulted in a significant increase in survival during the 4 weeks following treatment (Figure 2E).

### HMGB1 induces cardiac regeneration in failing hearts

**Figure 3:**
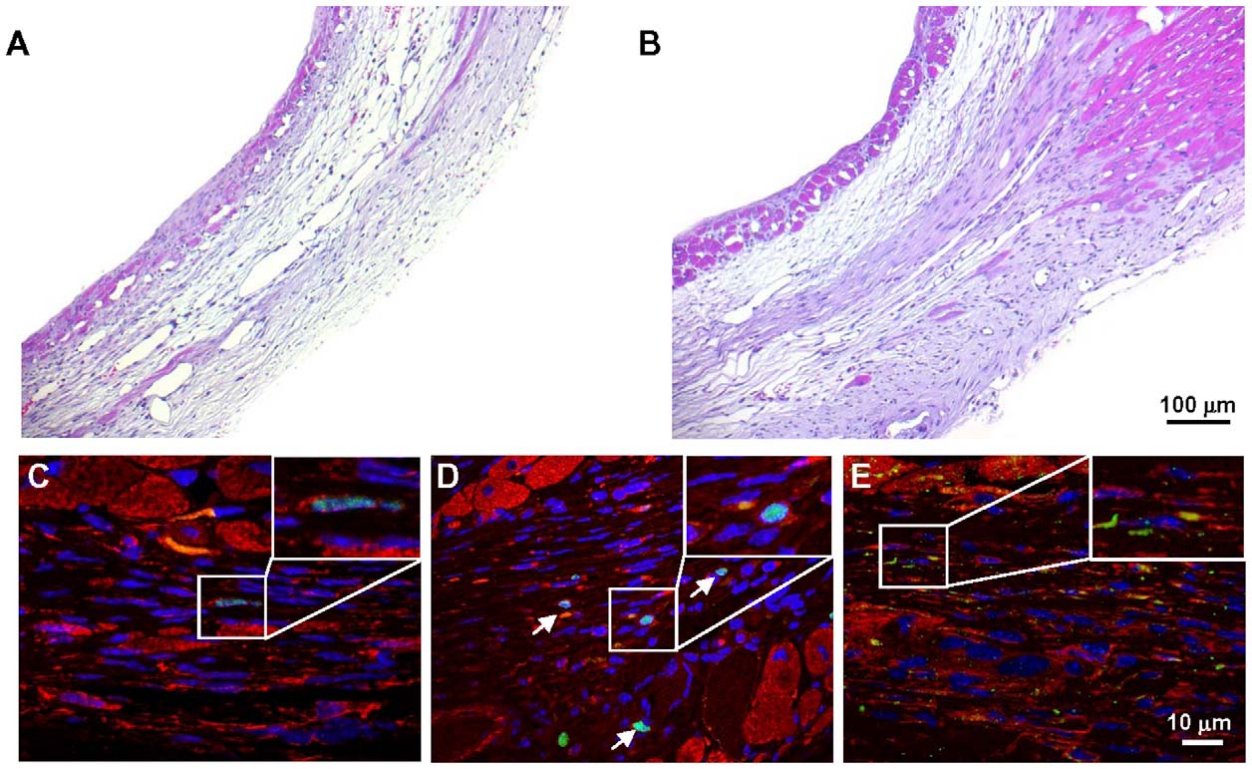
Newly formed cardiomyocytes in the failing heart. (A,B) Representative H&E stained myocardial sections of the infarcted region from control (A) and HMGB1-treated mice (B). (C–E) Regenerated myocytes in the infarcted region of HMGB1-treated hearts expressed α-sarcomeric actin (red fluorescence) in the cytoplasm, the nuclear protein Ki-67 (C) and evidence of BrdU incorporation (arrows) in the nucleus (D). Newly formed myocytes in the infarcted area were positive for connexin 43 (E; green fluorescence). Representative cells are shown at higher magnification in the inset. Nuclei are stained with DAPI.

**Figure 4:**
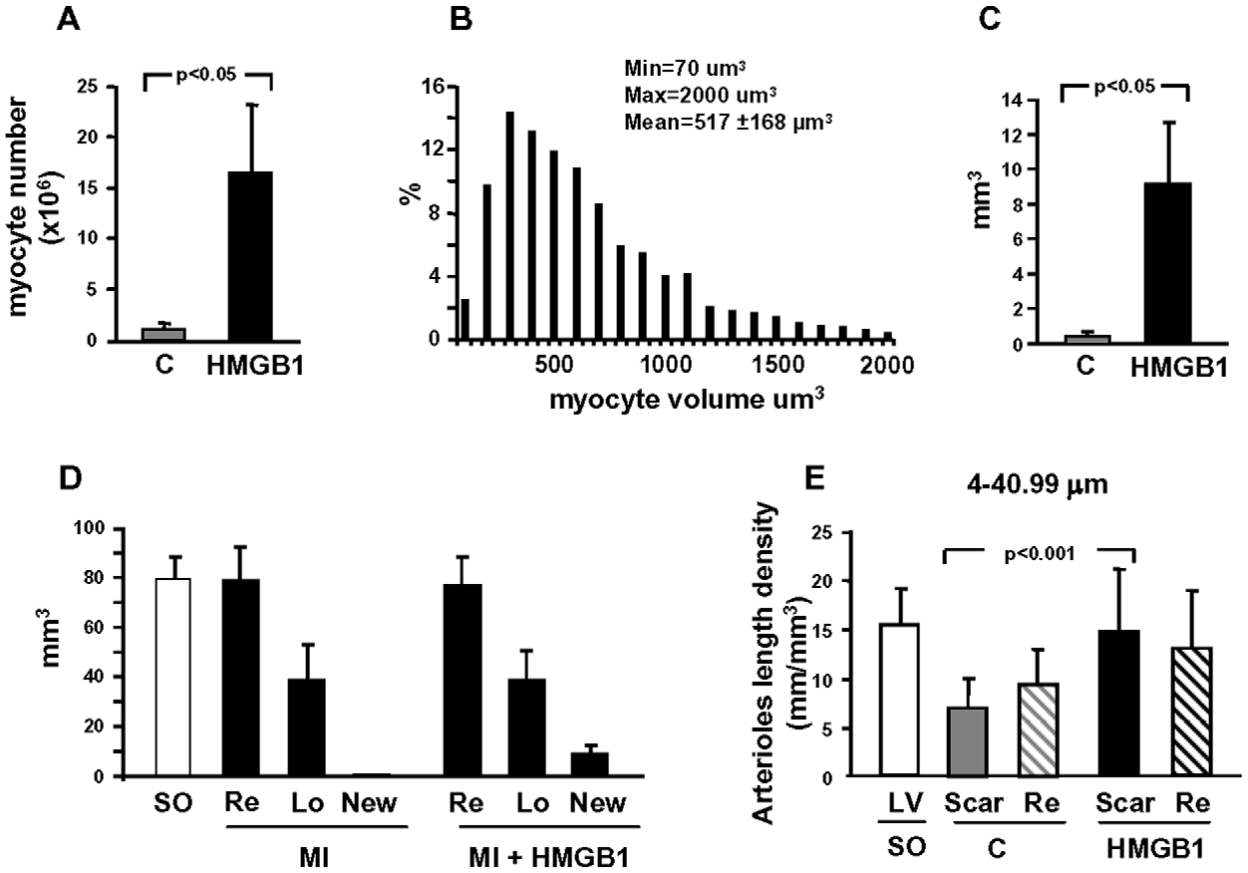
Cardiac regeneration in failing hearts. (A) Average number and (B) volume distribution of regenerated myocytes (control, n = 11; HMGB1, n = 13). (C) Average new myocardium volume in HMGB1-treated and control hearts (n = 13). (D) Reduction of infarct size by tissue regeneration: myocardium volume of the left ventricle in SO mice (n = 10) and volume of remaining (Re), lost (Lo) and regenerated (New) myocardium in HMGB1-treated (n = 13) and control (n = 11) animals. (E) Average arteriole length density (diameter 4–41 µm) in the scar tissue and in the remaining (Re) myocardium of control (C; n = 10) and HMGB1-treated (HMGB1; n = 10) hearts. Arteriole length density was also evaluated in the LV of SO mice. (n = 10). All measurements were performed 4 weeks after HMGB1 or control treatment.

We have previously shown that HMGB1 injected into the mouse LV wall four hours after coronary artery ligation induces c-kit^+^ cell activation, myocardial regeneration and improvement in cardiac function [5]. Here we examined whether HMGB1 induced myocardial regeneration also in the failing heart. In agreement with our previous work we found that 3 days after HMGB1 injection into the failing ventricle, the number of c-kit^+^ cells in the infarcted area increased from 1.17 to 3.18 cells/mm^2^ (p<0.05). Four weeks after treatment the infarcted region exhibited a thicker wall (Figures 3A and B) with a mixture of scar tissue and new myocardium, as demonstrated by the presence of a thin band of small cells expressing the cardiac marker α-sarcomeric actin, Ki67, a protein present in the nucleus of cycling cells (Figure 3C) and BrdU incorporation (Figure 3D). Further, these small cardiomyocytes expressed connexin 43, a protein responsible for electrical coupling among myocytes. This protein was present in the cytoplasm and at the surface of closely aligned differentiating cells demonstrating that the new myocardial cells were acquiring functional competence (Figure 3E). Treated hearts generated an average 16.5 million new myocytes (Figure 4A) that had volumes varying from 70 to 2000 µm^3^ (Figure 4B) and were localized within the infarcted area; altogether regeneration accounted for 9±3.7 mm^3^ of new myocardium (Figure 4C) and resulted in 24±5% recovery of scar tissue (Figure 4D). Since cell survival is dependent upon blood flow, we also quantified arterioles length density in the scar and in the non-infarcted left ventricle. It has been previously demonstrated that HMGB1 induces angiogenesis, both *in vitro* and *in vivo*, in several animal models of tissue damage [11], [13], [14]. In agreement with those studies we found that, four weeks after HMGB1 administration, the length density of arterioles 4 to 41 µm in diameter, detected by anti-α-smooth muscle actin immunostaining, was significantly enhanced in the scar region of the treated group compared to the control group (Figure 4E); indeed, in the infarcted region there was a significant decrease in arterioles and HMGB1 enhanced arteriole length density to a level comparable to that in sham operated mice. It is noteworthy that the increase in arteriole 4 to 21 µm in diameter accounted for this response and that the trend increase in the length density of arterioles 21 to 41 µm in diameter failed to achieve statistical significance (Figure S3).

### HMGB1 reduces collagen deposition and enhances collagenolytic activity in the infarcted region

**Figure 5:**
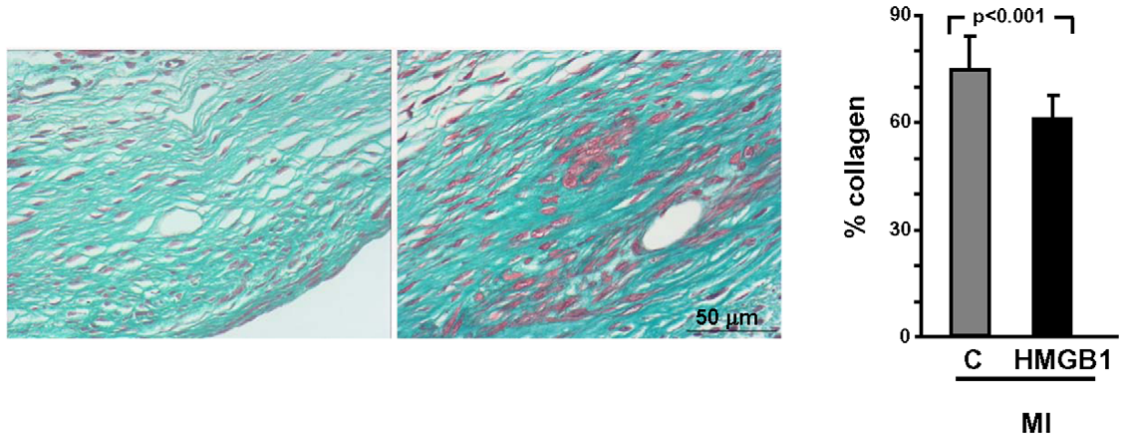
Collagen in the failing heart. (A) Representative Masson's Trichrome staining photomicrographs of control (C) and HMGB1-treated (HMGB1) hearts, 4 weeks after treatment. (B) Average results show a lower collagen content in HMGB1-treated (HMGB1; n = 14) than in control (C; n = 10) hearts.

**Figure 6:**
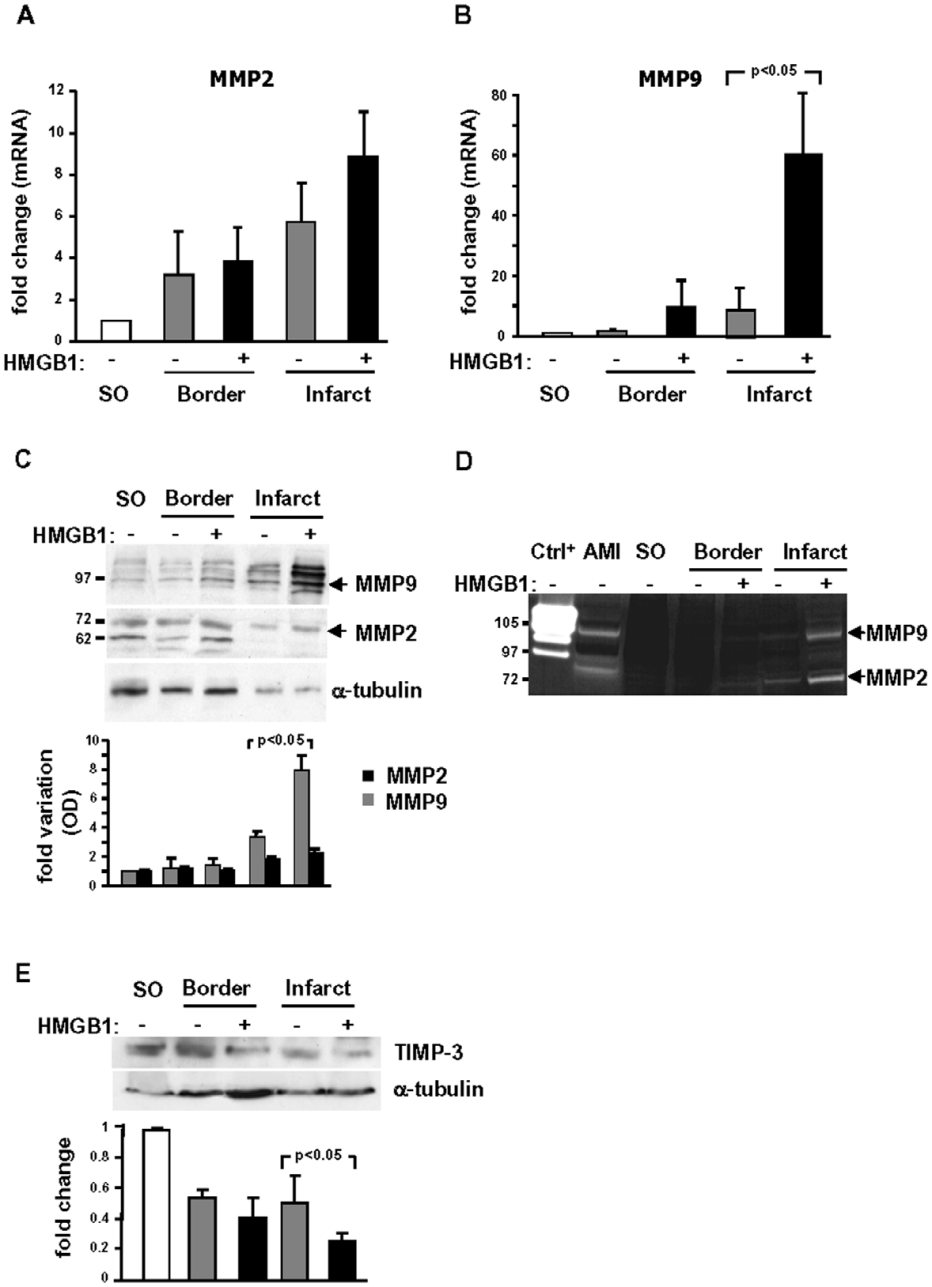
MMP-2, MMP-9, TIMP-3 expression and collagenolytic activity in the failing heart. All experiments depicted in this figure were performed three days after HMGB1 or control treatment. Both MMP-2 (A) and MMP-9 (B) mRNAs increased in the infarcted heart; HMGB1 had no effect on MMP-2 expression (n = 5) whereas it enhanced MMP-9 expression (n = 5), both in the border zone and in the infarcted area. (C) HMGB1 increased MMP-9 protein in the infarcted area whereas it had no significant effect on MMP-2 protein. (D) HMGB1 markedly increased MMP-2 and MMP-9 gelatinase activity in the infarcted area vs control hearts. This experiment was repeated 3 times with similar results. (E) TIMP-3 protein levels were lower in failing hearts, both in the border zone and in the infarcted area, than in SO hearts. HMGB1 caused a further decrease in TIMP-3 protein. Both for MMPs (C) and TIMP-3, the same filter was probed with α-tubulin to normalize protein loading (n = 5 for the average results of densitometric analyses of western blots).

In light of the effect of HMGB1 on LV volume and remodelling, and of the significant recovery of scar tissue, we quantified collagen deposition in the scar by Masson's Trichrome staining and quantitative digital image analysis. We found that, four weeks after treatment, collagen density was 14% lower in HMGB1-treated than in control hearts (Figure 5). In order to account for this result it was evaluated whether HMGB1 had an effect on the expression of matrix metalloproteinases (MMPs) and their inhibitors (TIMPs), and whether collagenolytic activity was modulated. Failing hearts expressed MMP-2 and MMP-9 (Figures 6A and 6B); HMGB1 administration had no significant effect on MMP-2 mRNA and protein levels (Figures 6A and 6C) whereas it markedly enhanced MMP-9 mRNA and protein (Figures 6B and 6C). Further, both MMP-2 and MMP-9 collagenolytic activity increased in the infarcted area of HMGB-1 treated hearts (Figure 6D). Since among the four known TIMPs only TIMP-3 inhibits both MMP-2 and MMP-9 activity [15], we examined the protein level of this inhibitor; we found that TIMP-3 in the border zone and in the infarcted area was lower than in the left ventricle of sham operated hearts and that HMGB1 caused a further decrease in TIMP-3 protein (Figure 6E). In contrast, there was no effect of HMGB1 on TIMP-4 expression (Figure S4).

### HMGB1 induces miR-206 expression *in vivo* and *in vitro*

**Figure 7:**
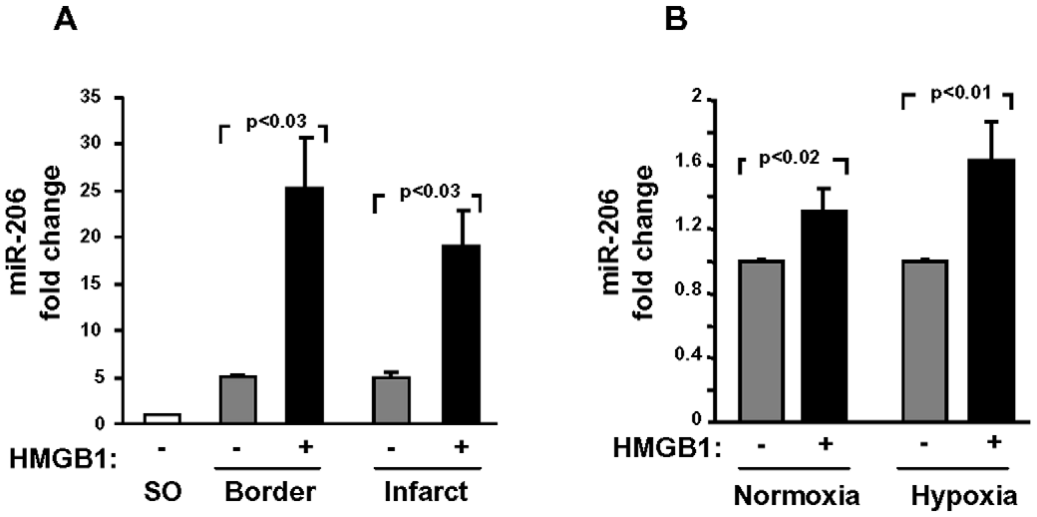
HMGB1 enhances miR-206 expression in failing hearts and in cardiac fibroblasts. (A) qRT-PCR analysis of miR-206 expression in the border zone and in the infarcted area of HMGB1-treated and control hearts 3 days after treatment, as well as in the left ventricle of SO mice (n = 5/group). In failing hearts, three weeks after MI, there was a significant increase in miR-206 expression, both in the border zone and in the infarcted area; HMGB1 had an additional effect to markedly enhance miR-206 expression. (B) qRT-PCR analysis of miR-206 expression in 24 hr normoxic and hypoxic cultured CFs. HMGB1 (100 ng/ml) enhanced miR-206 expression both in normoxia and hypoxia (n = 3/group).

Several miRNA have been involved in the regulation of myocardial cell proliferation and differentiation, cytoskeletal organization, cardiac hypertrophy and fibrosis [16], [17]. Therefore, it was examined whether, under our experimental conditions, HMGB1 had an effect on miRNAs known to play an important role in modulating cardiac structure and function. In agreement with prior studies in animal models of heart failure it was found that miR-21 and miR-208b increased both in the infarct area and in the border zone, miR-1 and miR-208a decreased in the infarct area, miR-133a decreased in the border zone whereas the trend decrease in the infarct area did not achieve statistical significance, and miR-133b exhibited only a non-significant diminution both in the infarct and in the border zone [18]–[22] (Figure S5). Further, although miR-29 family has been involved in post-MI fibrosis through the regulation of collagen [23], no significant expression changes were observed in our experimental model between healthy and failing hearts (Figure S5). None of these miRNAs was significantly modulated by HMGB1 except for miR-133b further diminution in the infarct area of HMGB1-treated hearts (Figure S5). In contrast, muscle specific miR-206, which has been previously shown to increase acutely after MI [24], was markedly up-regulated in failing hearts (Figure 7A) and, following treatment with HMGB1, it exhibited a ∼4–5-fold additional increase, both in the border zone and in the infarct area (Figure 7A), which represented a ∼20–25 fold increase vs the LV expression level in sham operated mice Additional experiments were performed *in vitro* to confirm HMGB1 effect on miR-206 expression. Cardiac fibroblasts (CFs) were isolated from adult mouse hearts and cultured for 24 hr under normoxic or hypoxic conditions, either in the presence or absence of HMGB1. Both in normoxia and hypoxia, HMGB1 enhanced miR-206 expression and this response was more pronounced at low oxygen concentration (Figure 7B).

### miR-206 down-modulates TIMP-3 expression

**Figure 8:**
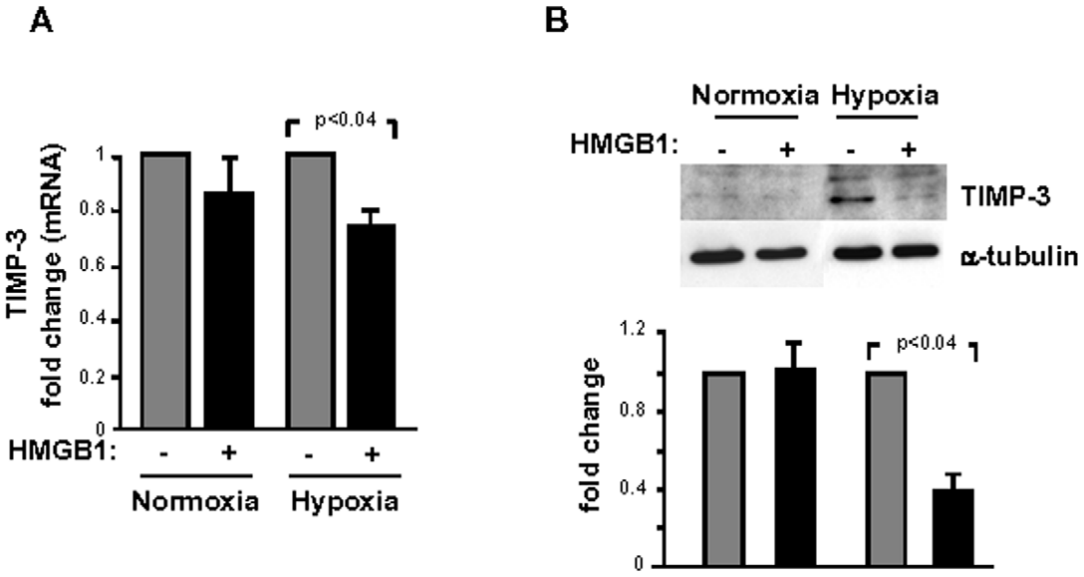
HMGB1 reduces TIMP3 in cardiac fibroblasts. CFs were cultured under normoxic or hypoxic conditions for 24 hrs, either in the presence or absence of 100 ng/ml HMGB1. Under hypoxic conditions both TIMP-3 mRNA (A) and protein (B) diminished upon exposure to HMGB1 (n = 3/group).

Since HMGB1 up-regulates miR206 expression, we sought to clarify the possible functions of this miRNA in HMGB1-mediated effects in failing hearts. A bioinformatics-based approach was employed to predict the putative mRNA targets containing evolutionarily conserved miR-206 seed match sequences in their 3′UTRs [25]–[27]. Notably, among potential candidates we identified TIMP-3 which we had found down-modulated in failing hearts following HMGB1 administration (Figure 6E). The *in vitro* experiments were performed in CFs isolated from mouse hearts and cultured either in normoxic or in hypoxic conditions. CFs expressed MMP-2 and MMP-9 and HMGB1 administration to the culture medium enhanced both MMPs expression in hypoxic cells (Figure S6). This effect was paralleled by a significant decrease of TIMP-3 mRNA and protein levels under hypoxia (Figures 8A and 8B).

**Figure 9:**
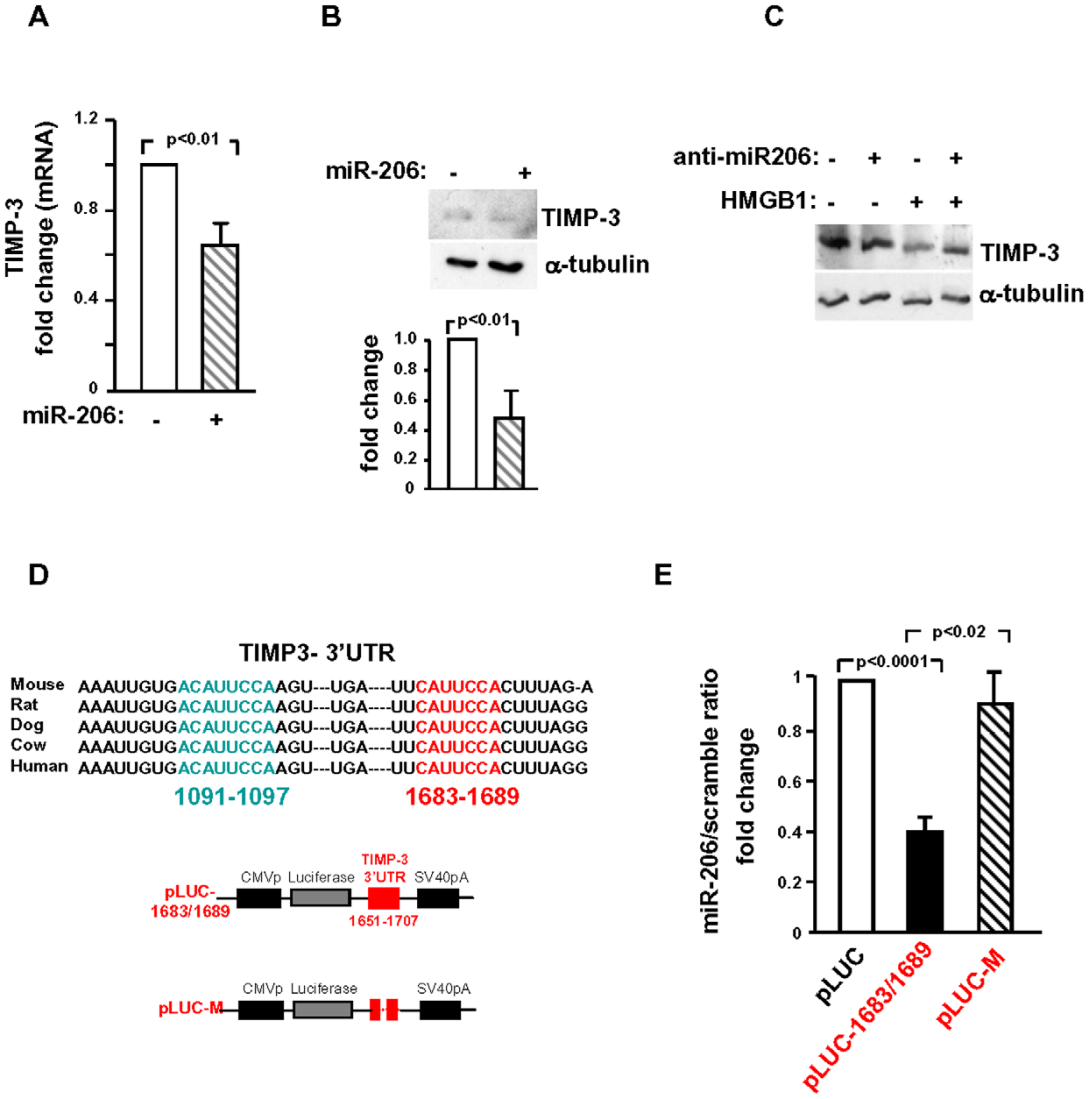
miR-206 targets TIMP-3. Lentivirus-mediated miR-206 overexpression in hypoxic CFs inhibited TIMP-3 mRNA (A) and protein (B) levels. TIMP-3 mRNA and protein were analyzed 3 and 24 hr after infection, respectively. Average results of densitometric analyses of western blot are also shown. (C) Representative western blot shows that the effect of HMGB1 to decrease TIMP-3 protein in hypoxic CFs was rescued by anti-miR-206. Cells were transfected with anti-miR206 and exposed to hypoxia for 24 hr either in the presence or in the absence of 100 ng/ml HMGB1. For western blot analysis (B,C), the same filter was probed with α-tubulin to normalize protein loading. (D) Upper panel: Conservation of miR-206 seed match sequences (in red and blue) in mammalian TIMP-3. Lower panel: Diagram of plasmid construction; the TIMP-3-3′UTR containing the 1683-1689 seed sequence (1651–1707) or the respective mutated segment were cloned downstream of the luciferase encoding sequence. (E) HEK293 cells were transfected with vector alone (pLUC) or firefly luciferase constructs that contain either the intact (pLUC-1683–1689) or the mutated (pLUC-M) miR-206 binging site. Each plasmid was cotransfected with a plasmid encoding Renilla luciferase along with miR-206 or scramble sequence. Firefly luciferase values were normalized to Renilla luciferase activity and the ratio of luciferase activity of each construct in the presence and in the absence of miR206 was calculated. Luciferase activity decreased upon pLUC-1683–1689 and miR-206 transfection whereas it was not modulated by cotransfection of pLUC-M and miR-206 (n = 6/group). These results indicate that miR-206 binds the 1683–1689 seed sequence in TIMP-3-3′UTR.

To corroborate these results, we performed miR-206 gain- and loss-of-function experiments and measured TIMP-3. Overexpression of miR-206 in normoxic conditions significantly reduced TIMP-3 mRNA (Figure 9A) and protein levels (Figure 9B). Further, knockdown of endogenous miR-206 by anti-miR-206 counteracted HMGB1-mediated down-regulation of TIMP-3 protein levels (Figure 9C). In order to establish whether miR-206 targets TIMP-3 mRNA directly, a luciferase reporter assay was performed. Two complementary base pair matching sequences for miR-206 were located at 1090–1097 and at 1683–1689 of the TIMP-3-3′UTR. Interestingly, both seed sequences of TIMP-3-3′UTR to miR-206 are highly conserved among species and are identical in mouse, rat, dog, cow and human (Figure 9D). To validate whether miR-206 recognizes the 3′UTR of TIMP-3, two constructs containing each miR-206 seed sequence and the immediately surrounding sequences in TIMP-3 were cloned downstream of the luciferase open reading frame; pLUC-1090/97 and pLUC-1683/89 (Figure 9E and data not shown). The luciferase activity of each construct was evaluated following their cotransfection in HEK-293 cells either with miR-206 or a negative control miRNA. Luciferase activity from pLUC-1683/89 was markedly inhibited by miR-206 overexpression whereas this effect was prevented by the deletion of the seed complementary nucleotides in pLUC-M (Figure 9E); in contrast, miR-206 seed-pairing site at position 1090/97 was not functional in the assayed conditions (data not shown). Thus, the seed sequence 1683/89 represents the specific target of miR-206 and is responsible for miR-206-mediated TIMP-3 down-regulation.

## Discussion

In the present study we demonstrated that HMGB1 injected into murine chronically failing hearts enhanced LV function and attenuated LV remodelling; these effects were associated with myocardial and vascular regeneration, increased MMP-2 and MMP-9 activity, miR-206 overexpression and miR-206 -mediated inhibition of TIMP-3.

The effect of HMGB1 in animal models of cardiac ischemia has been previously examined in acute MI, ischemia/reperfusion injury and post-MI heart failure. Our laboratory has shown that HMGB1 administration, acutely after MI, induces cardiac progenitor cell activation, myocardial regeneration and an improvement in cardiac performance [5]. Further, it has been shown that coronary artery ligation in transgenic mice with cardiac-specific overexpression of HMGB1 resulted, four weeks after MI, in enhanced angiogenesis, improved survival and restored cardiac function [28]. In agreement with those results, HMGB1 blockade by administration of neutralizing anti-HMGB1 antibody in a rat model of MI worsened cardiac remodelling [29]. The effect of HMGB1 in ischemia/reperfusion injury has been addressed in three separate studies and the results have been controversial. The systemic administration of an anti-HMGB1 antibody to block endogenous HMGB1 worsened cardiac damage [30] and exogenous HMGB1 administered *via* the perfusate to the whole heart in a Langendorff preparation had a beneficial effect [31]. In contrast, another study reported that exogenous HMGB1 administered intraperitoneally worsened cardiac damage whereas the opposite result was achieved by endogenous HMGB1 inhibition with the intraperitoneal injection of HMGB1 boxA [32]. Finally, two prior studies have examined the effects of HMGB1 in failing hearts [33], [34]. Volz et al. showed that HMGB1-specific blockage significantly reduced post-myocardial infarction remodeling. Notably, this effect was not detected in mice lacking the HMGB1 receptor RAGE. Takahashi et al. utilized a rat model of post-MI heart failure and an experimental protocol similar to the one adopted in the present work, i.e. three weeks after coronary ligation HMGB1 was injected intramyocardially at a dose that, adjusted by weight, was similar to the dose injected in our protocol; thereafter mice were followed for four additional weeks. Interestingly, it was found an improvement in ejection fraction and a decrease in extracellular collagen deposition; those results were confirmed in the present study. However, differently from Takahashi et al, in our work we also demonstrated an increase in newly formed myocardial cells and an increase in arteriole length density in the infarcted area. Further, we found a marked increase in MMP-2 and MMP-9 activity and a decrease in TIMP-3 expression. No prior study has shown an effect of HMGB1 on MMP-2 and TIMP-3 whereas it has been recently reported that HMGB1 enhances MMP-9 expression in neurons, *via* Toll-Like Receptor 4 signaling [35], and in cancer cells *via* NF-κB signalling [36]. Indeed, NF-κB is the intracellular target of HMGB1 and its activation results in cell migration [37] and in the modulation of the inflammatory response [38]. It is noteworthy that enhanced MMPs activity may favour the migration of activated resident progenitor cells into the scar and cardiac regeneration as it has been shown to occur in response to the intramyocardial injection of hepatocyte growth factor (HGF) and insulin-like growth factor-1 (IGF-1)[6]. Activated MMPs can be directly inhibited by interaction with TIMPs resulting in the prevention of matrix degradation [39]. TIMP-3 is highly expressed in the heart and inhibits different MMPs including MMP-2 and MMP-9; further, it differs from other TIMPs because it binds ECM which may lead to prolonged bioavailability, localized action and a major effect on LV remodelling and fibrosis. Indeed, TIMP-3 levels are significantly reduced in patients with dilated cardiomyopathy and heart failure [40]. The present work confirms that in the failing heart TIMP-3 expression is lower than in control and also shows that HMGB1 consistently causes a further decrease in TIMP-3 expression, both *in vivo*, in the infarcted area, and *in vitro*, in hypoxic CFs. Inhibition of TIMP-3 expression is expected to contribute to the increase in MMP-2 and MMP-9 activities and the decrease in collagen content in HMGB1-treated failing hearts. However, there are also important MMPs-independent effects of TIMP-3 that should be considered. TIMP-3 inhibits neonatal cardiomyocyte proliferation [15] and myogenic differentiation of muscle stem cells [41], enhances CFs activation, phenotypic differentiation toward myofibroblasts and BrDU incorporation [42], promotes smooth muscle cell apoptosis [15] and inhibits VEGF-induced angiogenesis *in vitro* and *in vivo* [43]. Further, the hearts of TIMP-3-deficient mice after MI [44], [45] exhibit decreased collagen content and increased blood vessel density. Notably, HMGB1 also enhances VEGF release from CFs [46], suggesting that multiple mechanisms may be involved in the angiogenic response to HMGB1 reported in the present and other studies[10], [13], [47], [11]. Thus, TIMP-3 inhibition may account for different HMGB1 effects in the failing heart; enhanced MMP-2 and MMP-9 activity that leads to a decrease in collagen in the failing left ventricle, enhanced angiogenesis and increased cardiac stem cell proliferation and differentiation that ultimately leads to myocardial regeneration.

Recent reports have shown the involvement of a variety of miRNAs in cardiac disease and, specifically, in cardiac remodelling and fibrosis [17], however, under our experimental conditions HMGB1 did not modulate these miRNA. In contrast, miR-206 increased in the failing LV and HMGB1 had a marked effect to further increase miR-206 expression, both in the border zone and in the infarcted area. In agreement with this result, HMGB1 induced miR-206 upregulation also *in vitro,* in cultured CFs. Interestingly miR-206 has been extensively characterized in skeletal muscle development; it plays a major role in muscle differentiation [48] and is highly expressed in regenerating muscle fibers, both in Duchenne Muscle Dystrophy and several days after acute hindlimb ischemia [49]. Further, miR-206 overexpression in C2C12 myoblasts has been recently shown to downregulate TIMP-3 mRNA [41]. Regarding the heart, it has been shown only that miR-206 increases after MI [24] but its functional role is unexplored. In light of the major effect of HMGB1 on miR-206 we performed gain- and loss-of-function studies, established that miR-206 modulates TIMP-3 mRNA and protein and, in a luciferase reporter assay, we identified the seed sequence 1683/89 as the specific target of miR-206 responsible for TIMP-3 down-regulation.

In conclusion, here we provide new insights into the mechanisms by which HMGB1 induces myocardial regeneration, new blood vessel development, prevents remodelling and improves function in the failing heart. Importantly, miR-206-mediated down-regulation of TIMP-3 may underlie some of the cardiac effects of exogenous HMGB1 described in prior studies and in the present work.

## Materials and Methods

All experimental procedures in mice complied with the Guidelines of the Italian National Institutes of Health, with the Guide for the Care and Use of Laboratory Animals (Institute of Laboratory Animal Resources, National Academy of Sciences, Bethesda, MD, USA) and were approved by the Institutional Animal Care and Use Committee. The approval number is MM39.

An expanded Materials and Methods section containing details for in vivo studies, cell isolation and culture, immunohistochemistry, western blot, gel zymography, mRNA and miRNA isolation and amplification can be found in Materials and Methods S1.

### miR-206 Overexpression and Down-modulation

To overexpress miR-206, miR-206 mimic or a control scramble sequence (Table S1, Applied Biosystems) were transfected by small interfering RNA transfection reagent (Santa Cruz Biotechnology) in 70% confluent cardiac fibroblasts at the final concentration of 40 and 240 nM for mir-206 overexpression and inhibition, respectively. To block endogenous miR-206, locked Nucleic Acid oligonucleotides against miR-206 or a control scramble sequence were transfected using the same protocol. After 16 h, cells were re-fed with fresh medium, maintained either in normoxia or exposed to hypoxia and experiments were performed up to 24 h later.

### Reporter plasmid generation, expression and luciferase assay

A luciferase reporter vector (pMir-Report; Ambion, Inc, Austin, Tex. USA) was used to generate the luciferase constructs. Oligonucleotides bearing wt or deleted miR-206-seed pairing sites of TIMP-3 gene downstream of the stop codon (Table S1) were cloned in pMIR-REPORT-Luciferase (pLUC, Ambion Inc.), between SpeI and Hind III restriction sites. The oligonucleotides used are listed in Table S1. The PCR products were then digested with *HindIII* and *SPEI*, and the fragment was inserted into a *HindIII* and *SPEI* -digested pMir-Report luciferase plasmid, to obtain a luciferase construct pLuc1683/89 and pLuc 1090/97. Mutant construct were generated using similar approach.

HEK-293 cells, plated in 24 well-plate, were transfected with 100 ng of pLUC, pLuc1683/89, pLuc 1090/97 pLuc1683/89M and pLuc 1090/97M, 10 pmol of miR-206 scramble, and 2.5 ng of pRL-null renilla luciferase. Cellular extracts were tested with Dual Luciferase Assay (Promega, Milan, IT), according to the manufacturer instructions, 48 hrs after transfection, using a Synergy HT luminometer (BioTek Instruments, Winooski, VT USA). Values were normalized according to renilla luciferase and the ratio of firefly luciferase of each construct was calculated either in the presence or in the absence of exogenous miR-206.

### Data collections and Statistics

Results are presented as mean±standard error unless otherwise indicated. Statistical significance between two measurements was evaluated by unpaired Student's *t* test and multiple comparisons was performed by Bonferroni method [12]. A probability value of p<0.05 was considered significant.

## Supporting Information

Figure S1 **Experimental protocol.** Myocardial infarction (MI) was induced in mice by coronary artery ligation. After 2 weeks, echocardiographic measurements were performed to evaluate LV function and size. One week later, HMGB1 or denatured HMGB1 (control; C) was injected in the peri-infarct area. Echocardiography was repeated 2 weeks after injection (5 weeks after MI) and, again 4 weeks after injection (7 weeks after MI); the last echocardiogram was followed by hemodynamic evaluation, thereafter mice were sacrificed.
(TIF)

Figure S2 **Hemodynamic assessment of cardiac function.** (A) LV+dP/dt (rate of pressure rise) and (B) LV -dP/dt (rate of pressure decay) in sham operated (SO), control (C) and HMGB1-treated infarcted mice (MI). Measurements were obtained 4 weeks after HMGB1 treatment (7 weeks after MI) (Results are presented as mean±standard deviation; SO, n = 10; control, n = 14; HMGB1, n = 19).
(TIF)

Figure S3 **HMGB1 enhances arteriole density in failing hearts.** (A) Representative photomicrograph of an arteriole in the infarcted region of a HMGB1-treated heart, 4 weeks after treatment; the arteriole is stained with a α-smooth muscle actin antibody. (B–D) Bar graph showing arteriole length density. Arterioles were grouped according to their diameter (4–10.99 µm; 11–20.99 µm; 21–40.99 µm) and were quantified in the scar tissue and in the remaining myocardium (Re) of control (C; n = 10) and HMGB1-treated (HMGB1; n = 10) hearts as well as in the LV of SO mice (n = 10).
(TIF)

Figure S4 **HMGB1 does not modulate TIMP-4 expression.** HMGB1 was injected into the LV three weeks after MI; three days after HMGB1 injection it was found no modulation of TIMP-4 mRNA expression vs control. Values in bar graphs are reported as fold change vs control (n = 3/group).
(TIF)

Figure S5 **Effect of HMGB1 on cardiac miRNAs expression.** HMGB1 was injected into the LV three weeks after MI; three days after HMGB1 injection the expression of the indicated miRNAs was evaluated by qRT-PCR both in the border zone and in the infarcted area. Values are reported as fold change vs SO hearts (n = 5/group).
(TIF)

Figure S6 **Effect of HMGB1 on MMP-2 and MMP-9 expression in cultured cardiac fibroblasts.** MMP2 (A) and MMP9 (B) mRNA levels were determined in CFs cultured in normoxia or hypoxia and treated with HMGB1 (100 ng/ml) for 6 hr (n = 3/group). All values are reported as fold change vs control.
(TIF)

Table S1 **miRNA and mRNA.**
(PPT)

Materials and Methods S1 Details of in vivo studies including the heart failure animal model as well as the functional and histological evaluation of failing hearts, cell isolation and culture, gel zymography western blot and miRNA studies, were described in Materials and Methods S1.
(DOC)

## References

[1] Krum H (2009). Optimising management of chronic heart failure.. Lancet.

[2] Orlic D, Kajstura J, Chimenti S, Jakoniuk I, Anderson SM (2001). Bone marrow cells regenerate infarcted myocardium.. Nature.

[3] Bearzi C, Rota M, Hosoda T, Tillmanns J, Nascimbene A (2007). Human cardiac stem cells.. Proc Natl Acad Sci U S A.

[4] Urbanek K, Rota M, Cascapera S, Bearzi C, Nascimbene A (2005). Cardiac stem cells possess growth factor-receptor systems that after activation regenerate the infarcted myocardium, improving ventricular function and long-term survival.. Circ Res.

[5] Limana F, Germani A, Zacheo A, Kajstura J, Di Carlo A (2005). Exogenous high-mobility group box 1 protein induces myocardial regeneration after infarction via enhanced cardiac C-kit+ cell proliferation and differentiation.. Circ Res.

[6] Rota M, Padin-Iruegas ME, Misao Y, De Angelis A, Maestroni S (2008). Local activation or implantation of cardiac progenitor cells rescues scarred infarcted myocardium improving cardiac function.. Circ Res.

[7] Germani A, Limana F, Capogrossi MC (2007). Pivotal advances: high-mobility group box 1 protein--a cytokine with a role in cardiac repair.. J Leukoc Biol.

[8] Ding HS, Yang J (2010). High mobility group box-1 and cardiovascular diseases.. Saudi Med J.

[9] Palumbo R, Sampaolesi M, De Marchis F, Tonlorenzi R, Colombetti S (2004). Extracellular HMGB1, a signal of tissue damage, induces mesoangioblast migration and proliferation.. J Cell Biol.

[10] Chavakis E, Hain A, Vinci M, Carmona G, Bianchi ME (2007). High-mobility group box 1 activates integrin-dependent homing of endothelial progenitor cells.. Circ Res.

[11] De Mori R, Straino S, Di Carlo A, Mangoni A, Pompilio G (2007). Multiple Effects of High Mobility Group Box Protein 1 in Skeletal Muscle Regeneration Arterioscler Thromb Vasc Biol.

[12] Anversa P, Olivetti G (2002). The cardiovascular system: the heart Handbook of Physiology Oxford University Press, New York.

[13] Mitola S, Belleri M, Urbinati C, Coltrini D, Sparatore B (2006). Cutting edge: extracellular high mobility group box-1 protein is a proangiogenic cytokine.. J Immunol.

[14] Schlueter J, MaÌnner J, Brand T (2006). BMP is an important regulator of proepicardial identity in the chick embryo.. Developmental Biology.

[15] Vanhoutte D, Heymans S (2009). TIMPs and cardiac remodeling: ‘Embracing the MMP-independent-side of the family’.. J Mol Cell Cardiol.

[16] Barringhaus KG, Zamore PD (2009). MicroRNAs: regulating a change of heart.. Circulation.

[17] Small EM, Frost RJ, Olson EN (2010). MicroRNAs add a new dimension to cardiovascular disease.. Circulation.

[18] Care A, Catalucci D, Felicetti F, Bonci D, Addario A (2007). MicroRNA-133 controls cardiac hypertrophy.. Nat Med.

[19] Ikeda S, He A, Kong SW, Lu J, Bejar R (2009). MicroRNA-1 negatively regulates expression of the hypertrophy-associated calmodulin and Mef2a genes.. Mol Cell Biol.

[20] Thum T, Gross C, Fiedler J, Fischer T, Kissler S (2008). MicroRNA-21 contributes to myocardial disease by stimulating MAP kinase signalling in fibroblasts.. Nature.

[21] van Rooij E, Sutherland LB, Qi X, Richardson JA, Hill J (2007). Control of stress-dependent cardiac growth and gene expression by a microRNA.. Science.

[22] Satoh M, Minami Y, Takahashi Y, Tabuchi T, Nakamura M (2010). Expression of microRNA-208 is associated with adverse clinical outcomes in human dilated cardiomyopathy.. J Car Fail.

[23] van Rooij E, Sutherland LB, Thatcher JE, DiMaio JM, Naseem RH (2008). Dysregulation of microRNAs after myocardial infarction reveals a role of miR-29 in cardiac fibrosis.. Proc Natl Acad Sci U S.

[24] Shan ZX, Lin QX, Fu YH, Deng CY, Zhou ZL (2009). Upregulated expression of miR-1/miR-206 in a rat model of myocardial infarction.. Biochem Biophys Res Commun.

[25] Betel D, Wilson M, Gabow A, Marks DS, Sander C (2008). The microRNAorg resource: targets and expression.. Nucleic Acids Res.

[26] Krek A, Grun D, Poy MN, Wolf R, Rosenberg L (2005). Combinatorial microRNA target predictions.. Nat.

[27] Lewis, BP Burge, CB Bartel, DP (2005). Conserved seed pairing, often flanked by adenosines, indicates that thousands of human genes are microRNA targets.. Cell.

[28] Kitahara T, Takeishi Y, Harada M, Niizeki T, Suzuki S (2008). High-mobility group box 1 restores cardiac function after myocardial infarction in transgenic mice.. Cardiovasc Res.

[29] Kohno T, Anzai T, Naito K, Miyasho T, Okamoto M (2009). Role of high-mobility group box 1 protein in post-infarction healing process and left ventricular remodelling.. Cardiovasc Res.

[30] Oozawa S, Mori S, Kanke T, Takahashi H, Liu K (2008). Effects of HMGB1 on ischemia-reperfusion injury in the rat heart.. Circ J.

[31] Abarbanell AM, Hartley JA, Herrmann JL, Weil BR, Wang Y (2011). Exogenous high-mobility group box 1 improves myocardial recovery after acute global ischemia/reperfusion injury.. Surgery.

[32] Andrassy M, Volz HC, Igwe JC, Funke B, Eichberger SN (2008). High-mobility group box-1 in ischemia-reperfusion injury of the heart.. Circulation.

[33] Takahashi K, Fukushima S, Yamahara K, Yashiro K, Shintani Y (2008). Modulated inflammation by injection of high-mobility group box 1 recovers post-infarction chronically failing heart.. Circulation.

[34] Volz HC, Seidel C, Laohachewin D, Kaya Z, Muller OJ (2010). HMGB1: the missing link between diabetes mellitus and heart failure.. Basic Res Cardiol.

[35] Qiu J, Xu J, Zheng Y, Wei Y, Zhu X (2010). High-mobility group box 1 promotes metalloproteinase-9 upregulation through Toll-like receptor 4 after cerebral ischemia.. Stroke.

[36] Liu PL, Tsai JR, Hwang JJ, Chou SH, Cheng YJ (2009). High-mobility group box 1-mediated matrix metalloproteinase-9 expression in non-small cell lung cancer contributes to tumor cell invasiveness.. Am J Respir Cell Mol Biol.

[37] Palumbo R, Galvez BG, Pusterla T, De Marchis F, Cossu G (2007). Cells migrating to sites of tissue damage in response to the danger signal HMGB1 require NF-kappaB activation.. J Cell Biol.

[38] Ulloa L, Messmer D (2006). High-mobility group box 1 (HMGB1) protein: friend and foe.. Cytokine Growth Factor Rev.

[39] Creemers EE, Cleutjens JP, Smits JF, Daemen MJ (2001). Matrix metalloproteinase inhibition after myocardial infarction: a new approach to prevent heart failure?. Circ Res.

[40] Li YY, Feldman AM, Sun Y, McTiernan CF (1998). Differential expression of tissue inhibitors of metalloproteinases in the failing human heart.. Circulation.

[41] Liu H, Chen SE, Jin B, Carson JA, Niu A (2010). TIMP3: a physiological regulator of adult myogenesis.. J Cell Sci.

[42] Lovelock JD, Baker AH, Gao F, Dong JF, Bergeron AL (2005). Heterogeneous effects of tissue inhibitors of matrix metalloproteinases on cardiac fibroblasts.. Am J Physiol Heart Circ Physiol.

[43] Qi JH, Ebrahem Q, Moore N, Murphy G, Claesson-Welsh L (2003). A novel function for tissue inhibitor of metalloproteinases-3 (TIMP3): inhibition of angiogenesis by blockage of VEGF binding to VEGF receptor-2.. Nat Med.

[44] Tian H, Cimini M, Fedak PW, Altamentova S, Fazel S (2007). TIMP-3 deficiency accelerates cardiac remodeling after myocardial infarction.. J Mol Cell Cardiol.

[45] Kang KH, Park SY, Rho SB, Lee JH (2008). Tissue inhibitor of metalloproteinases-3 interacts with angiotensin II type 2 receptor and additively inhibits angiogenesis.. Cardiovasc Res.

[46] Rossini A, Zacheo A, Mocini D, Totta P, Facchiano A (2008). HMGB1-stimulated human primary cardiac fibroblasts exert a paracrine action on human and murine cardiac stem cells.. J Mol Cell Cardiol.

[47] Schlueter C, Weber H, Meyer B, Rogalla P, Roser K (2005). Angiogenetic signaling through hypoxia: HMGB1: an angiogenetic switch molecule.. Am J Pathol.

[48] Kim HK, Lee YS, Sivaprasad U, Malhotra A, Dutta A (2006). Muscle-specific microRNA miR-206 promotes muscle differentiation.. J Cell Biol.

[49] Greco S, De Simone M, Colussi C, Zaccagnini G, Fasanaro P (2009). Common micro-RNA signature in skeletal muscle damage and regeneration induced by Duchenne muscular dystrophy and acute ischemia.. Faseb J.

