# Supplementary figures and images for: HMGB1 Attenuates Cardiac Remodelling in the Failing Heart via Enhanced Cardiac Regeneration and miR-206-Mediated Inhibition of TIMP-3
Source: PLoS One. 2011 Jun 22;6(6):e19845. doi: 10.1371/journal.pone.0019845 (PMC3120764; doi:10.1371/journal.pone.0019845)

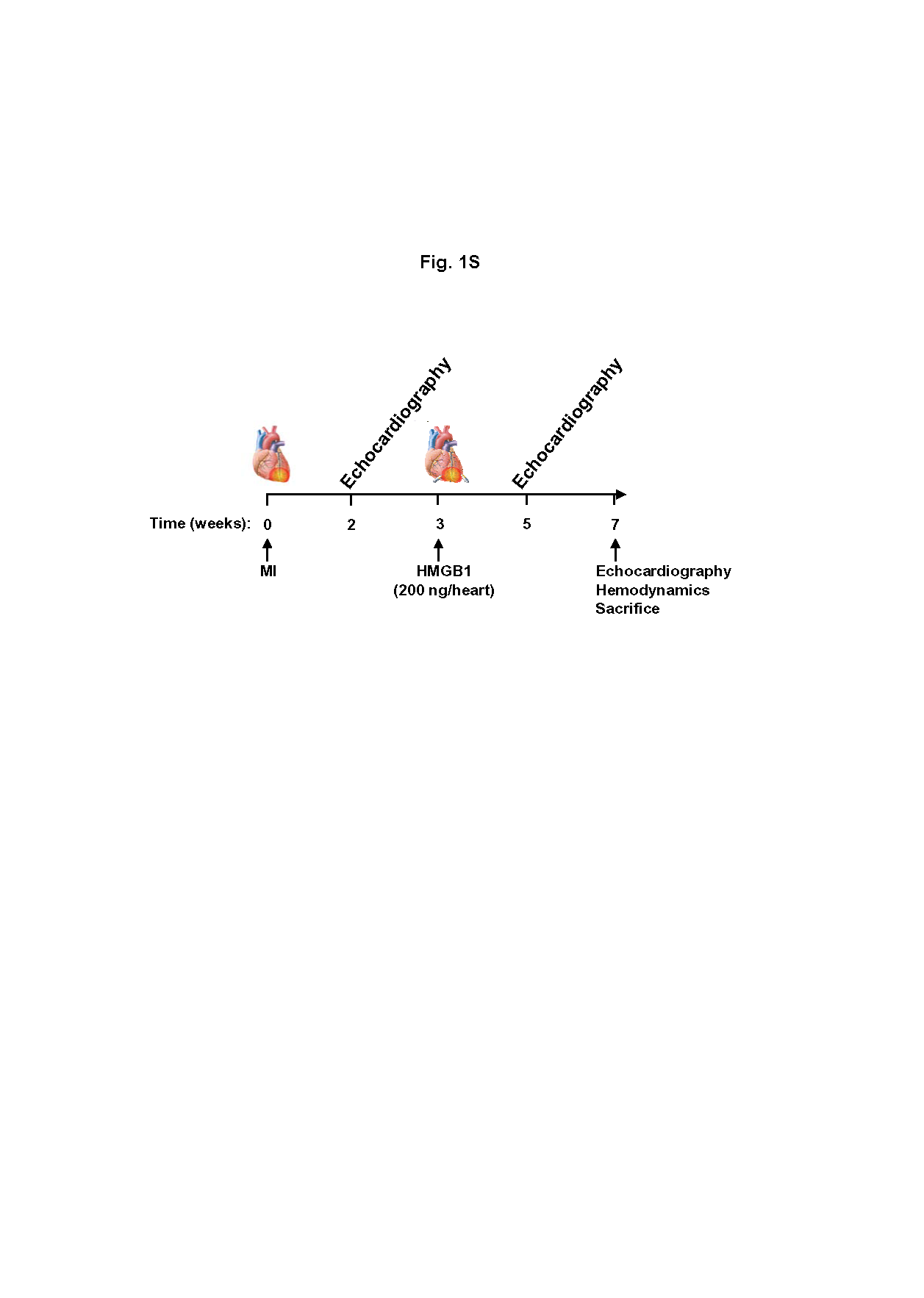

Supplement: Figure S1 — Experimental protocol. Myocardial infarction (MI) was induced in mice by coronary artery ligation. After 2 weeks, echocardiographic measurements were performed to evaluate LV function and size. One week later, HMGB1 or denatured HMGB1 (control; C) was injected in the peri-infarct area. Echocardiography was repeated 2 weeks after injection (5 weeks after MI) and, again 4 weeks after injection (7 weeks after MI); the last echocardiogram was followed by hemodynamic evaluation, thereafter mice were sacrificed. (TIF) [file pone.0019845.s001.tif]

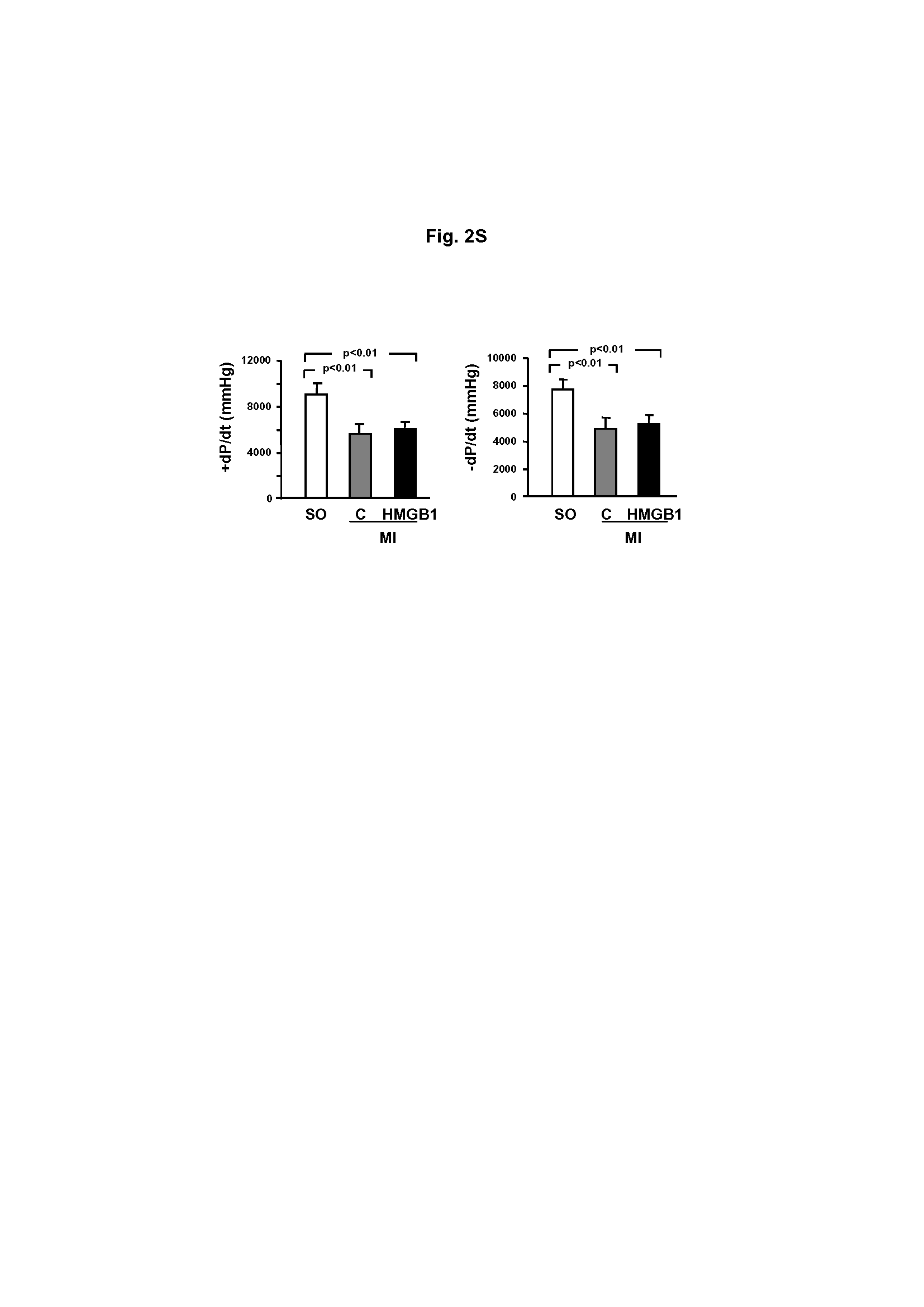

Supplement: Figure S2 — Hemodynamic assessment of cardiac function. (A) LV+dP/dt (rate of pressure rise) and (B) LV -dP/dt (rate of pressure decay) in sham operated (SO), control (C) and HMGB1-treated infarcted mice (MI). Measurements were obtained 4 weeks after HMGB1 treatment (7 weeks after MI) (Results are presented as mean±standard deviation; SO, n = 10; control, n = 14; HMGB1, n = 19). (TIF) [file pone.0019845.s002.tif]

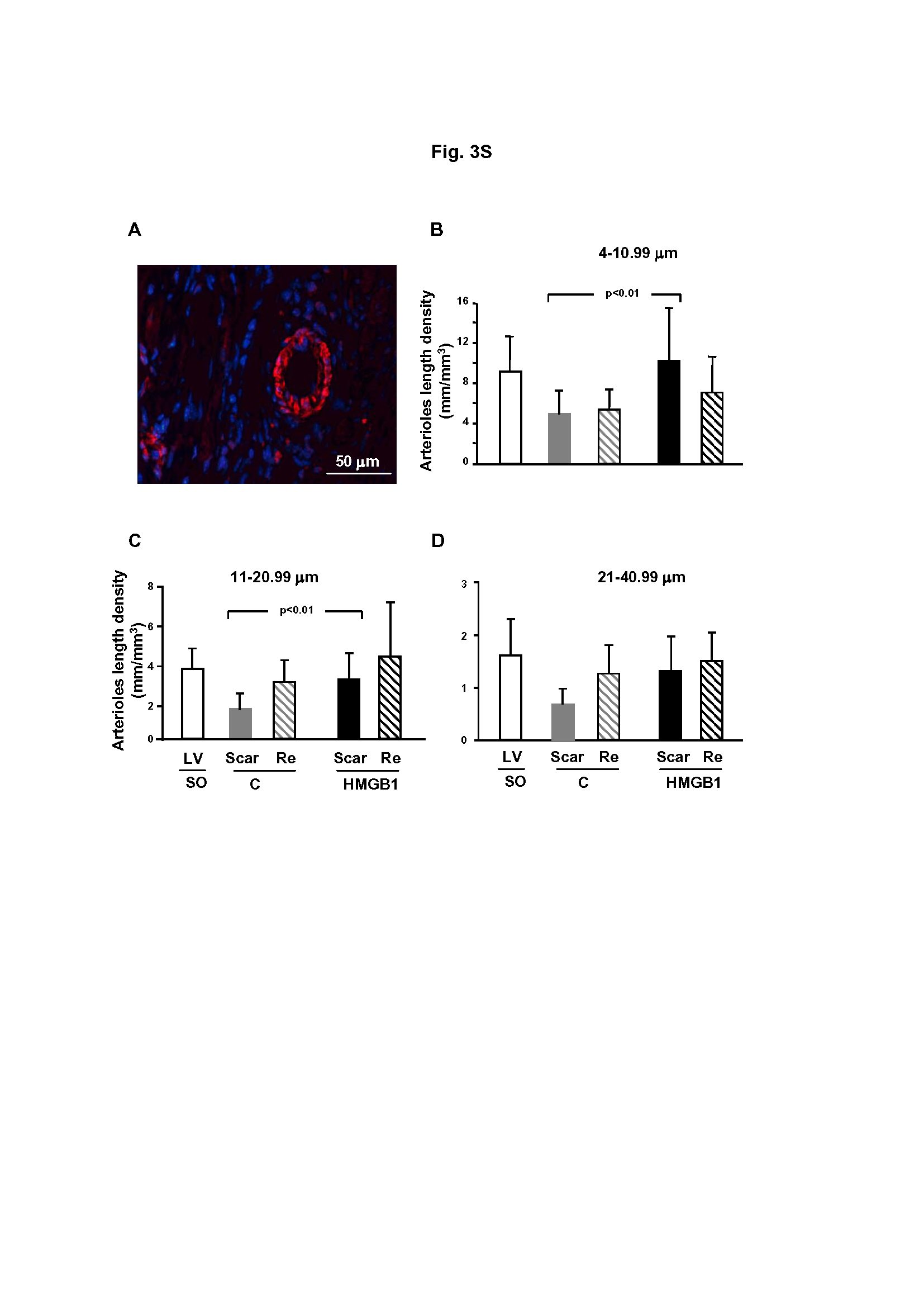

Supplement: Figure S3 — HMGB1 enhances arteriole density in failing hearts. (A) Representative photomicrograph of an arteriole in the infarcted region of a HMGB1-treated heart, 4 weeks after treatment; the arteriole is stained with a α-smooth muscle actin antibody. (B–D) Bar graph showing arteriole length density. Arterioles were grouped according to their diameter (4–10.99 µm; 11–20.99 µm; 21–40.99 µm) and were quantified in the scar tissue and in the remaining myocardium (Re) of control (C; n = 10) and HMGB1-treated (HMGB1; n = 10) hearts as well as in the LV of SO mice (n = 10). (TIF) [file pone.0019845.s003.tif]

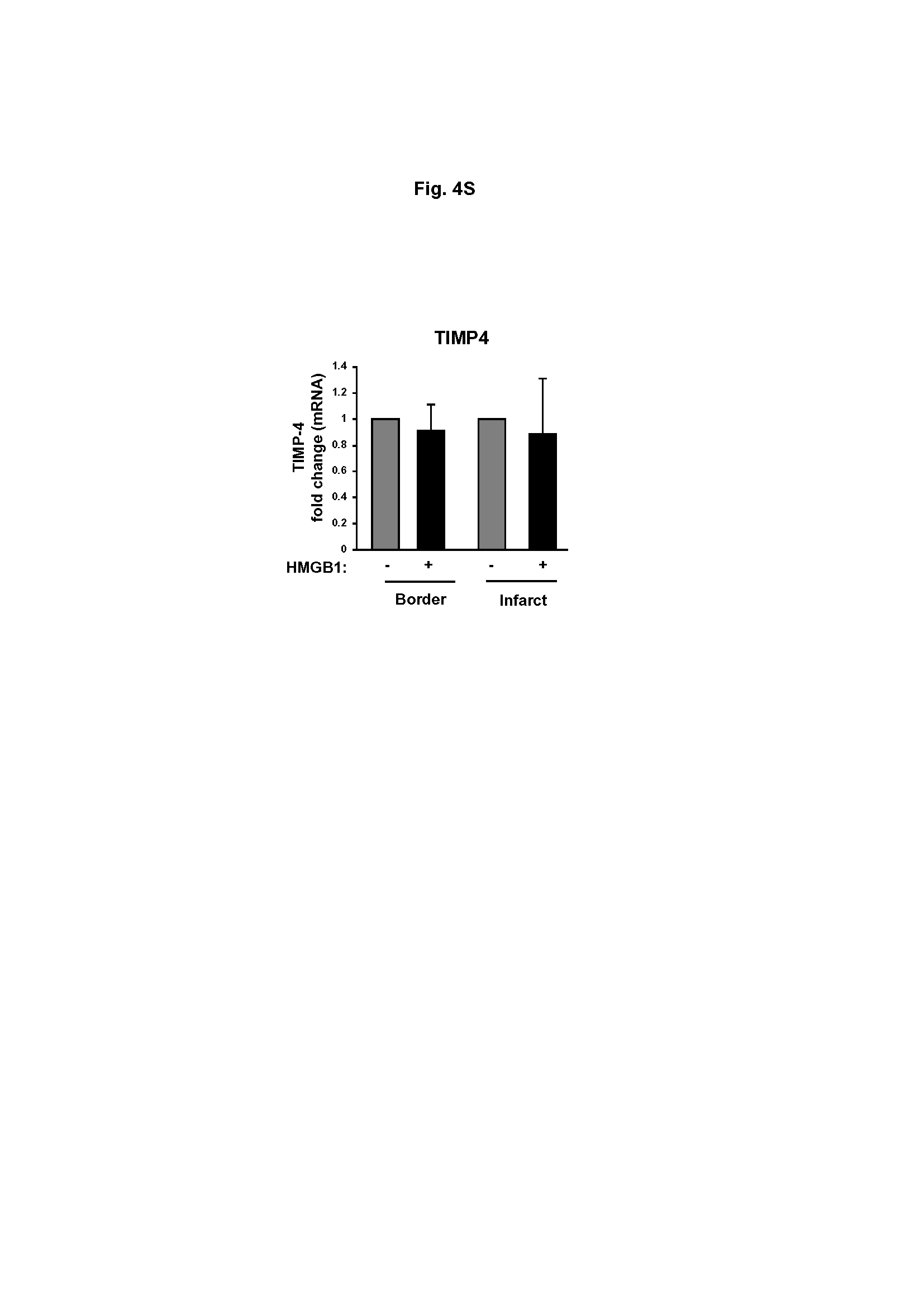

Supplement: Figure S4 — HMGB1 does not modulate TIMP-4 expression. HMGB1 was injected into the LV three weeks after MI; three days after HMGB1 injection it was found no modulation of TIMP-4 mRNA expression vs control. Values in bar graphs are reported as fold change vs control (n = 3/group). (TIF) [file pone.0019845.s004.tif]

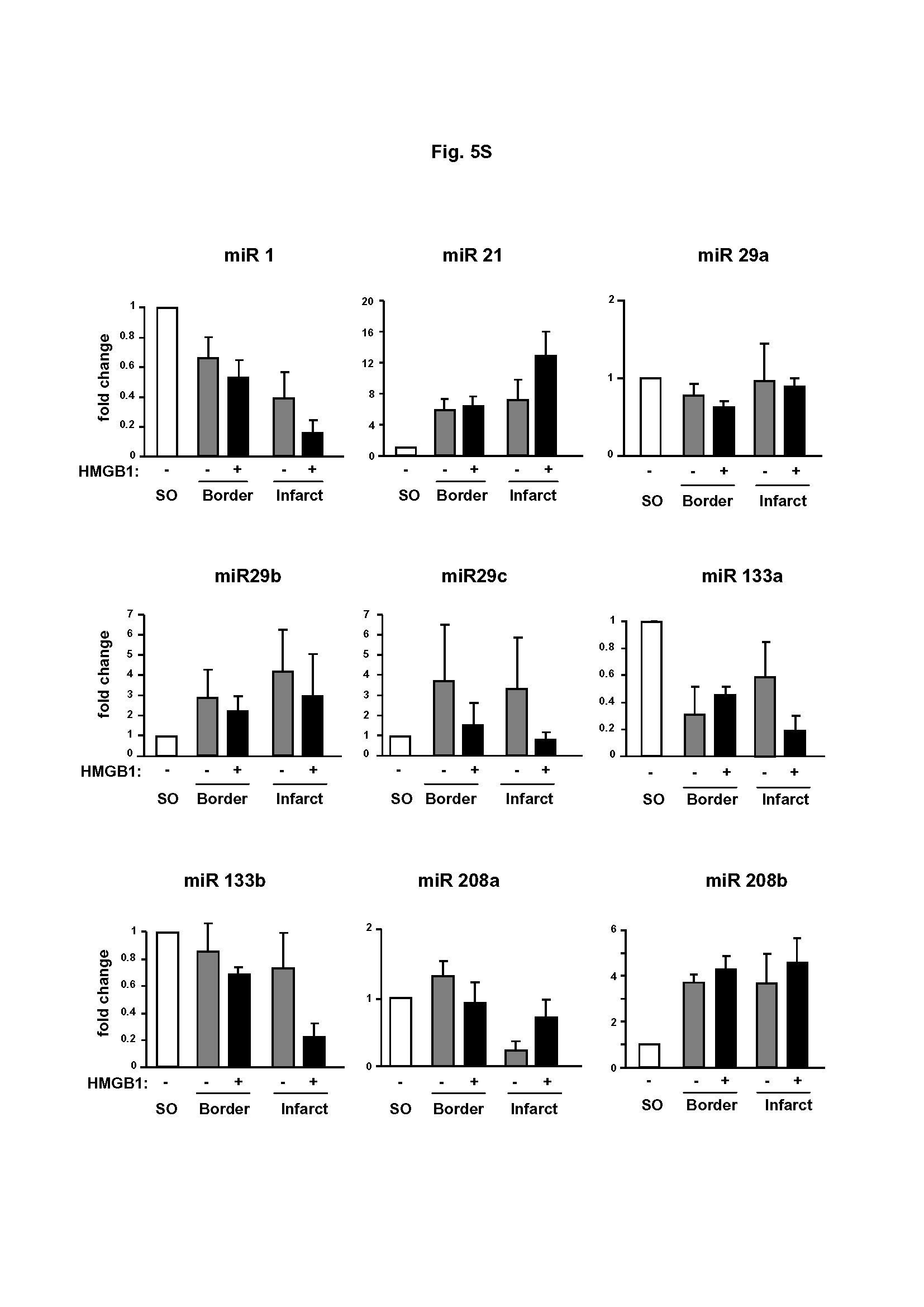

Supplement: Figure S5 — Effect of HMGB1 on cardiac miRNAs expression. HMGB1 was injected into the LV three weeks after MI; three days after HMGB1 injection the expression of the indicated miRNAs was evaluated by qRT-PCR both in the border zone and in the infarcted area. Values are reported as fold change vs SO hearts (n = 5/group). (TIF) [file pone.0019845.s005.tif]

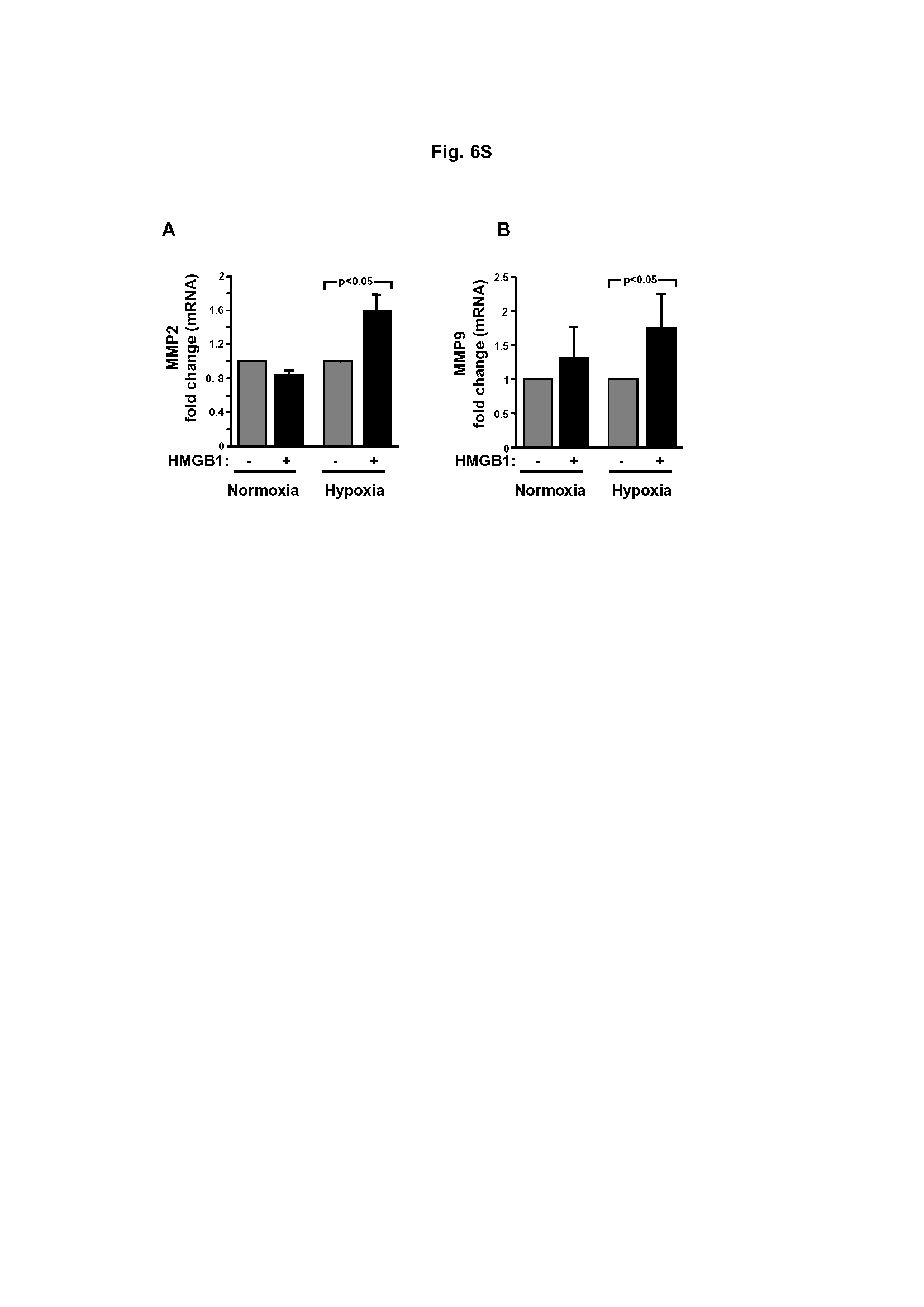

Supplement: Figure S6 — Effect of HMGB1 on MMP-2 and MMP-9 expression in cultured cardiac fibroblasts. MMP2 (A) and MMP9 (B) mRNA levels were determined in CFs cultured in normoxia or hypoxia and treated with HMGB1 (100 ng/ml) for 6 hr (n = 3/group). All values are reported as fold change vs control. (TIF) [file pone.0019845.s006.tif]
