# Supplementary material for: HMGB1 Attenuates Cardiac Remodelling in the Failing Heart via Enhanced Cardiac Regeneration and miR-206-Mediated Inhibition of TIMP-3
Source: PLoS One. 2011 Jun 22;6(6):e19845. doi: 10.1371/journal.pone.0019845 (PMC3120764; doi:10.1371/journal.pone.0019845)
Supplement: Table S1 — miRNA and mRNA. (PPT) [file pone.0019845.s007.ppt]

## Slide 1
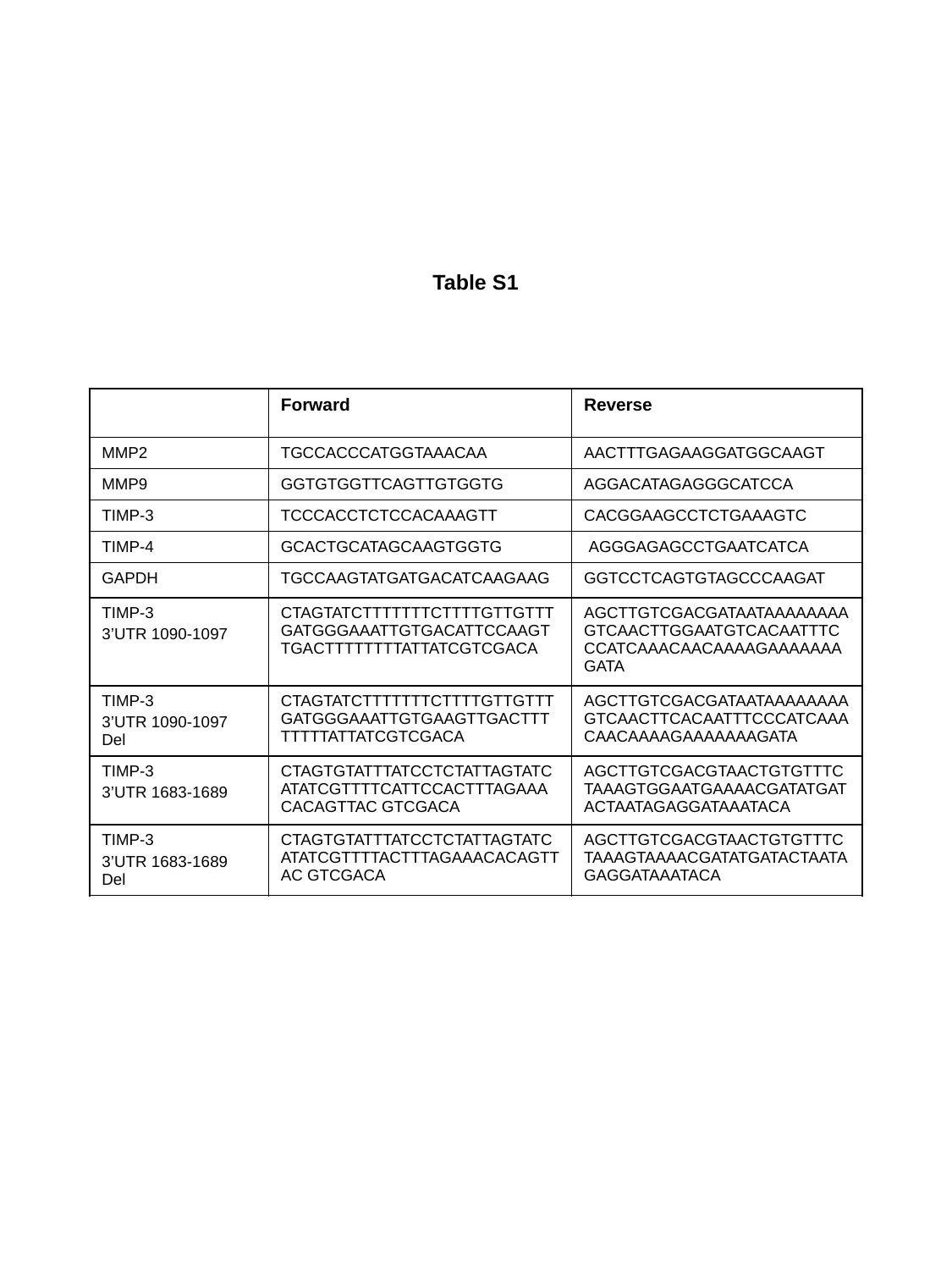

Table S1
| | Forward | Reverse |
| --- | --- | --- |
| MMP2 | TGCCACCCATGGTAAACAA | AACTTTGAGAAGGATGGCAAGT |
| MMP9 | GGTGTGGTTCAGTTGTGGTG | AGGACATAGAGGGCATCCA |
| TIMP-3 | TCCCACCTCTCCACAAAGTT | CACGGAAGCCTCTGAAAGTC |
| TIMP-4 | GCACTGCATAGCAAGTGGTG | AGGGAGAGCCTGAATCATCA |
| GAPDH | TGCCAAGTATGATGACATCAAGAAG | GGTCCTCAGTGTAGCCCAAGAT |
| TIMP-3 3’UTR 1090-1097 | CTAGTATCTTTTTTTCTTTTGTTGTTTGATGGGAAATTGTGACATTCCAAGTTGACTTTTTTTTATTATCGTCGACA | AGCTTGTCGACGATAATAAAAAAAAGTCAACTTGGAATGTCACAATTTCCCATCAAACAACAAAAGAAAAAAAGATA |
| TIMP-3 3’UTR 1090-1097 Del | CTAGTATCTTTTTTTCTTTTGTTGTTTGATGGGAAATTGTGAAGTTGACTTTTTTTTATTATCGTCGACA | AGCTTGTCGACGATAATAAAAAAAAGTCAACTTCACAATTTCCCATCAAACAACAAAAGAAAAAAAGATA |
| TIMP-3 3’UTR 1683-1689 | CTAGTGTATTTATCCTCTATTAGTATCATATCGTTTTCATTCCACTTTAGAAACACAGTTAC GTCGACA | AGCTTGTCGACGTAACTGTGTTTCTAAAGTGGAATGAAAACGATATGATACTAATAGAGGATAAATACA |
| TIMP-3 3’UTR 1683-1689 Del | CTAGTGTATTTATCCTCTATTAGTATCATATCGTTTTACTTTAGAAACACAGTTAC GTCGACA | AGCTTGTCGACGTAACTGTGTTTCTAAAGTAAAACGATATGATACTAATAGAGGATAAATACA |
