# Supplementary material for: HMGB1 Attenuates Cardiac Remodelling in the Failing Heart via Enhanced Cardiac Regeneration and miR-206-Mediated Inhibition of TIMP-3
Source: PLoS One. 2011 Jun 22;6(6):e19845. doi: 10.1371/journal.pone.0019845 (PMC3120764; doi:10.1371/journal.pone.0019845)
Supplement: Materials and Methods S1 — Details of in vivo studies including the heart failure animal model as well as the functional and histological evaluation of failing hearts, cell isolation and culture, gel zymography western blot and miRNA studies, were described in Materials and Methods S1. (DOC) [file pone.0019845.s008.doc]

**Materials and Methods S1**

*HMGB1 production*

Expression and purification of both HMGB1 and its truncated form BoxA were performed by HMGBiotech (Milan, IT). Endotoxins were removed by passage through Detoxy-Gel columns (Pierce Biotechnology Inc., Rockford, IL, USA). Recombinant HMGB1 was diluted in PBS and stored at –80°C.

*Animal Model and in vivo study*

MI was induced by coronary artery ligation in C57 BL6 female mice 8 weeks old (20 gr body weight), as previously described (1, 2). After 3 weeks, animals were re-operated and 200 ng of purified HMGB1 in 10 μl PBS solution, was injected through a 32-gauge needle. Four injections (2.5 μl per injection) were made in the ventricular wall bordering the viable myocardium. Sham operated and infarcted mice injected with 200 ng of denatured HMGB1 were used as control. 5-Bromo-2′-deoxyuridine (BrdU, Sigma, St. Louis, MO, USA) was given twice a day (50 mg/kg b.w., i.p.) starting one week before sacrifice to identify newly formed cells [3, 4]. Animals were sacrified 4 weeks after treatment, i.e. 7 weeks after infarction. A scheme illustrating this protocol is illustrated in Figure S1.

*Evaluation of myocardial function*

Echocardiography was performed in conscious mice at 2,5 and 7 weeks after myocardial infarction with a Sequoia 256c equipped with a 13-MHz linear transducer. Two-dimensional images and M-mode tracings were recorded from the parasternal short axis view at the level of papillary muscle. From M-mode tracings, anatomical parameters in diastole and systole were obtained [1, 5]. For hemodynamic studies, mice were anesthetized with chloral hydrate (400 mg/kg body weight) and the right carotid artery cannulated with a microtip pressure transducer (1.4F, Millar Instruments, Houston, TX, USA). The catheter was advanced into the LV cavity for the evaluation of LV pressures and + and − dP/dt in the closed chest preparation [1, 5].

*Cardiac anatomy and infarct size*

After hemodynamic measurements, the abdominal aorta was cannulated, the heart was arrested in diastole and the left ventricular (LV) chamber was fixed at a pressure equal to the *in vivo* measured end-diastolic pressure. The LV intracavitary axis was measured, and three transverse slices from the base, mid-region and apex were embebbed in paraffin. The mid-section was used to measure LV thickness, chamber diameter and volume as described [1]. The chamber volume was calculated using the minimal and maximal luminal diameters at midregion with the longitudinal axis. Diastolic wall stress was determined from the wall thickness, chamber radius and left ventricular end diastolic pressure (LVEDP). In each LV cross section it was calculated the infarct length, by measuring the endocardial and epicardial surface length delimiting the infarcted region, and the total LV. To obtain the dimension of the infarct and the extent of recovery with time, we employed a methodology already described in the literature [6, 7]. Specifically the percentage of lost and remaining myocardium in untreated and treated infarcted mice was calculated by measuring the quotient between the number of left ventricular myocytes in sham-operated mice and in the two groups of infarcted mice, respectively. Tissue specimens were embedded in paraffin and sections were obtained for immunohistochemical studies.

*Volume and Number of regenerated myocytes*

The volume of regenerated myocardium was determined by measuring the product of the area occupied by the restored tissue and the section thickness in each section. Sections were stained either with α-sarcomeric actin (clone 5C5; Sigma) or α-myosin heavy chain (Sigma) and nuclei were counterstained with bisBenzimide (H33258, Sigma) in order to measure the volume of randomly sampled myocytes in each heart. Only longitudinally oriented cells with centrally located nuclei were included. The length and diameter across the nucleus were collected in each myocyte to compute cell volume, assuming a cylindrical shape. The total number of regenerated myocytes was determined by measuring the ratio of the total volume of tissue repair over the average volume of the regenerated myocytes [4, 8].

## *Immunohistochemical analysis*

Hearts were arrested in diastole with cadmium chloride (100 mmol/L), perfused retrogradely with 10% (vol/vol) formalin, embedded in paraffin and sectioned (3 μm thickness). The following antibodies were used to identify cardiac stem cells and to assess cardiac differentiation: mouse monoclonal c-kit antibody (R&D Systems, MN, USA), rabbit polyclonal connexin 43 antibody (Sigma), mouse anti α-sarcomeric actin antibody (clone 5C5; Sigma). Ki67 was detected with mouse polyclonal antibody (clone NCL, Novocastra Laboratories, UK) and BrdU with a rabbit polyclonal antibody (Roche Applied Sciences, IN, USA). Fluorescein isothiocyanate (FITC) conjugated goat anti-rabbit and tetramethylrhodamine-5-(and 6)-isothiocyanate (TRITC) conjugated goat anti-mouse (Sigma) were used as secondary antibodies (Jackson ImmunoResearch, UK) (1).

*Vessel density*

Th density per unit area of myocardium was measured after sections were stained for alpha-smooth muscle actin (Sigma) as described [9].

*Collagen deposition*

Sections were stained with Masson’s trichrome. Total surface area and interstitial spaces were measured with computer-assisted videodensitometry (IAS 2000, Delta Sistemi, Rome, IT). Collagen fraction was calculated by the percentage of the total surface area occupied by the interstitial space minus the percentage of the total surface area [10].

*Cell isolation and culture*

Cardiac fibroblasts were isolated from C57BL6 female mice at 2–3 months of age by collagen digestion (280 U/mL; Worthington, Lakewood, N.J., USA) through the coronary arteries and myocyte removal by centrifugation. Following further purification in the Lymphoprep^TM^ gradient mononuclear heart cells were plated in F12:DMEM 1:1 supplemented with 10% fetal bovine serum. Twenty-four hour later, floating cells were removed and adherent cells, mainly composed by fibroblasts [11], were cultured in the same medium. HMGB1 was added at the concentration of 100ng/ml.

Hypoxia was induced by culturing cells in airtight modular incubator chambers (Forma Scientific, Mountain View, CA, USA), infused for 20 min with 95% N_2_ plus 5% CO_2_ and incubated at 37°C. In these conditions, oxygen concentration was about ~1% [12, 13].

*Western blot analysis*

Cardiac tissue was homogenized in RIPA buffer containing 10 mmol/L Tris-HCl (pH 7.4), 150 mmol/L NaCl, 1% Nonidet P40, 1% deoxycolic acid, 0.1% sodium dodecyl sulfate (SDS), 10% glycerol and protease inhibitors (2 μg/mL leupeptin, 2 μg/mLaprotinin and 1 mmol phenylmethylsulfonyl fluoride). In vitro cultured cells were resuspended in the same buffer. Equal amounts of total cellular proteins (100 μg/lane) were resolved by 8% SDS-polyacrylamide gelelectrophoresis and transferred to nitrocellulose membrane (Amersham Pharmacia Biotech, Little Chalfont, UK). Membranes were probed with MMP2/9/13 rabbit polyclonal antibody (pAb), (Abcam Cambridge, MA, USA); TIMP-3/4 pAb (Abcam); Col4A1 and Col4A3 got pAB (Santa Cruz Biotechnology, Santa Cruz, CA, USA); α -tubulin monoclonal antibody (mAb) 0.1 μg/ml , Oncogene Science Inc., Cambridge, MA, USA) followed by horseradish peroxidase-coupled secondary antibodies and developed by a chemiluminescence-based detection system (ECL, Amersham, Milan, IT).

*Gel zymography*

Heart tissue samples were rapidly removed and snap frozen. Frozen tissue was mechanically homogenized on ice in lysis buffer (50 mmol/L Tri-HCl, pH7.4; 150 mmol/L NaCl; 5 mmol/L CaCl_2_; 1% Nonidet P40; 0.1% SDS) including protease inhibitors Equal amounts of total cellular proteins were separated by electrophoresis on precast gels (10% polyacrylammide minigels containing 0.1% gelatin gel; (Invitrogen, Carlsbad, CA, USA) with SDS Sample Buffer (Invitrogen). After electrophoresis, gels were incubated in Renaturatuing Buffer and Developing Buffer according to manufacturer instructions (Invitrogen). Staining was performed with 0.5% Comassie Blue R-250 in 30% methanol and 10% acetic acid and destained with several washes in 30% methanol and 10% acetic acid (14, 15).

*mRNA and miRNA isolation, Real time RT-PCR, miRNA target prediction*

Total RNA, including miRNAs, was extracted from both heart tissue and cultured cells, using the TRIzol reagent (Invitrogen) according to the manufacturer's instructions. First-strand cDNAs were synthesized using a mixture of oligo(dT)_12–18_ primers with Superscript reverse transcriptase (Invitrogen). MiRNA levels were analyzed using the TaqMan real-time PCR (qPCR) method (5 ng/assay). Quantitative Reverse-transcription Polymerase Chain Reaction (RT-PCR) analysis was carried out using Power SYBR Green PCR master mix (Qiagen, Life Science, Milan, IT) and quantified with the 7900HT Fast Real-Time PCR system (Applied Biosystems, Foster City, CA, USA). Primer sequences are reported in Table S1. The housekeeping gene encoding glyceraldehyde-3-phosphate dehydrogenase (GAPDH) was used as internal control for mRNA expression studies. MiR-16 was used to normalize miRNA expression. Relative expression was calculated using the comparative cycle threshold (Ct) method (2^−ΔΔCt^). Bioinformatic prediction of miR-206 targets was performed using Pic-Tar (version 2006) and Target Scan (version 4.2).

*References*

1. Limana F, Germani A, Zacheo A, Kajstura J, Di Carlo A, et al (2005) Exogenous high-mobility group box 1 protein induces myocardial regeneration after infarction via enhanced cardiac C-kit+ cell proliferation and differentiation. Circ Res 97:e73-83.

2. Limana F, Bertolami C, Mangoni A, Di Carlo A, Avitabile D, et al (2009) Myocardial infarction induces embryonic reprogramming of epicardial c-kit(+) cells: role of the pericardial fluid. J Mol Cell Cardiol 48:609-618

3. Urbanek K, Rota M, Cascapera S, Bearzi C, Nascimbene A, et al (2005) Cardiac stem cells possess growth factor-receptor systems that after activation regenerate the infarcted myocardium, improving ventricular function and long-term survival. Circ Res 97:663-673.

4. Rota M, Padin-Iruegas ME, Misao Y, De Angelis A, Maestroni S, et al (2008) Local activation or implantation of cardiac progenitor cells rescues scarred infarcted myocardium improving cardiac function. Circ Res 103:107-116

5. Limana F, Zacheo A, Mocini D, Mangoni A, Borsellino G, et al (2007) Identification of myocardial and vascular precursor cells in human and mouse epicardium. Circ Res 101:1255-1265

6. Anversa P, Olivetti G (2002) The cardiovascular system: the heart. Handbook of Physiology Oxford University Press, New York:75-144

7. Tillmanns J, Rota M, Hosoda T, Misao Y, Esposito G, et al (2008) Formation of large coronary arteries by cardiac progenitor cells. Proc Natl Acad Sci U S A 105:1668-1673

8. Olivetti G, Capasso JM, Meggs LG, Sonnenblick EH, Anversa P (1991) Cellular basis of chronic ventricular remodeling after myocardial infarction in rats Circ Res 68:856-869

9. Loud AV, Anversa P (1984) Morphometric analysis of biologic processes. Lab Invest 50:250-261

10. Liu YH, Yang XP, Sharov VG, Nass O, Sabbah HN, et al (1997) Effects of angiotensin-converting enzyme inhibitors and angiotensin II type 1 receptor antagonists in rats with heart failure. Role of kinins and angiotensin II type 2 receptors J Clin Invest 99:1926-1935

11. Burgess ML, Terracio L, Hirozane T, Borg TK (2002) Differential integrin expression by cardiac fibroblasts from hypertensive and exercise-trained rat hearts Cardiovasc Pathol 11:78-87

12. Stern MD, Chien AM, Capogrossi MC, Pelto DJ, Lakatta EG (1985) Direct observation of the "oxygen paradox" in single rat ventricular myocytes Circ Res 56:899-903

13. Stern MD, Silverman HS, Houser SR, Josephson RA, Capogrossi MC, et al (1988) Anoxic contractile failure in rat heart myocytes is caused by failure of intracellular calcium release due to alteration of the action potential. Direct observation of the "oxygen paradox" in single rat ventricular myocytes. Proc Natl Acad Sci U S A 85:6954-6958

14. Brown PD, Levy AT, Margulies IM, Liotta LA, Stetler-Stevenson WG (1990) Independent expression and cellular processing of Mr 72,000 type IV collagenase and interstitial collagenase in human tumorigenic cell lines Cancer Res 50:6184-6191

15. Sumii T, Lo EH (2002) Involvement of matrix metalloproteinase in thrombolysis-associated hemorrhagic transformation after embolic focal ischemia in rats Stroke 33:831-836
